# In-line multi-wavelength non-destructive pharma quality monitoring with ultrabroadband carbon nanotubes photo-thermoelectric imaging scanners

**DOI:** 10.1038/s41377-025-01957-0

**Published:** 2025-09-11

**Authors:** Miki Kubota, Yuya Kinoshita, Sayaka Hirokawa, Daiki Shikichi, Noa Izumi, Naoko Hagiwara, Daiki Sakai, Yuto Matsuzaki, Minami Yamamoto, Leo Takai, Yukito Kon, Yuto Aoshima, Raito Ota, Mitsuki Kosaka, Meiling Sun, Yukio Kawano, Kou Li

**Affiliations:** 1 Department of Electrical, Electronic, and Communication Engineering, Faculty of Science and Engineering, Chuo University, 1-13-27 Kasuga, Bunkyo-ku, Tokyo 112-8551 Japan; 2 Laboratory for Future Interdisciplinary Research of Science and Technology, Tokyo Institute of Technology, 2-12-1 Ookayama, Meguro-ku, Tokyo 152-8552 Japan; 3 Department of Electrical and Electronic Engineering, School of Engineering, Tokyo Institute of Technology, 2-12-1 Ookayama, Meguro-ku, Tokyo 152-8552 Japan; 4 National Institute of Informatics, 2-1-2 Hitotsubashi, Chiyoda-ku, Tokyo 101-8430 Japan; 5 Kanagawa Institute of Industrial Science and Technology, 705-1 Imaizumi, Ebina-shi, Kanagawa 243-0435 Japan

**Keywords:** Imaging and sensing, Carbon nanotubes and fullerenes, Optical sensors, Integrated optics, Photonic devices

## Abstract

While non-destructive in-line monitoring at manufacturing sites is essential for safe distribution cycles of pharmaceuticals, efforts are still insufficient to develop analytical systems for detailed dynamic visualisation of foreign substances and material composition in target pills. Although spectroscopies, expected towards pharma testing, have faced technical challenges in in-line setups for bulky equipment housing, this work demonstrates compact dynamic photo-monitoring systems by selectively extracting informative irradiation-wavelengths from comprehensive optical references of target pills. This work develops a non-destructive in-line dynamic inspection system for pharma agent pills with carbon nanotube (CNT) photo-thermoelectric imagers and the associated ultrabroadband sub-terahertz (THz)–infrared (IR) multi-wavelength monitoring. The CNT imager in the proposed system functions in ultrabroadband regions over existing sensors, facilitating multi-wavelength photo-monitoring against external sub-THz–IR-irradiation. Under recent advances in the investigation of functional optical materials (e.g., gallium arsenide, vanadium oxide, graphene, polymers, transition metal dichalcogenides), CNTs play advantageous leading roles in collectively satisfying informative efficient photo-absorption and solution-processable configurations for printable device fabrication into freely attachable thin-film imagers in pharma monitoring sites. The above non-destructive dynamic monitoring system maintains in-line experimental setups by integrating the functional thin-film imager sheets and compact multiple photo-sources. Furthermore, permeable sub-THz–IR-irradiation, which provides different transmittance values specific to non-metallic materials per wavelength or composition, identifies constituent materials for pharma agents themselves and concealed foreign substances in a non-contact manner. This work finally inspects invisible detailed features of pharma pills with the non-destructive in-line dynamic photo-monitoring system by incorporating performances of CNT imagers and compact optical setups.

## Abstract (web summary)

Freely attachable carbon nanotube terahertz–infrared imager sheet performs in-line, dynamic, and real-time detailed testing of pharmaceutic pill products in an on-site non-destructive monitoring manner.

## Graphical abstract

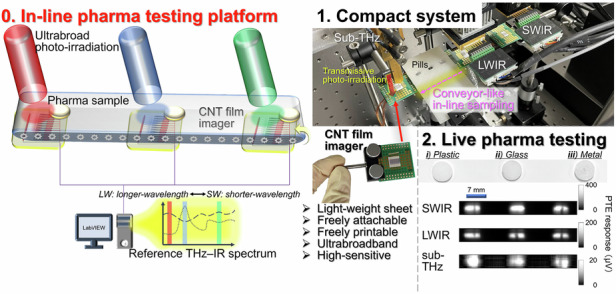

## Introduction

Non-destructive testing techniques for pharmaceuticals play an essential role in the current social systems^1,2^. Recent automation trends also contain the manufacturing and distribution of pharmaceuticals^3,4^. Therefore, hazardous defects concealed in a small part of pharmaceuticals potentially spread rapidly worldwide. From these viewpoints, reliable non-destructive pharma testing tools are essential. In connection with these, existing typical pharma testing methods are as follows: eyesight screening for irregular shapes^5,6^, partial spot-sampling for compositional agents^7,8^, and visible light (Vis) machine-learning for surface monitoring^9,10^. For safety cycles, non-destructive pharma testing specifically requires non-contact inner visualisation to target pills by analytical devices and systems. Furthermore, non-destructive material composition identification is essential for developing reliable pharma testing methods. This situation is mainly because most pharmaceuticals comprise multiple materials and layers (e.g., ingredients themselves and protection coating)^11,12^ as other daily necessities and industrial products^13,14^. In the above situation, non-destructive composition identification extracts detailed inspection cues, such as which constituent materials contain defects (e.g., cracks, breakages, and corrosion) or what kind of impurities target pills conceal. Therefore, non-destructive material composition identification specifies sources of abnormalities and minimises social distribution incidents of defective products.

To this end, longer-wavelength optical monitoring techniques^15–17^ have garnered another attention compared to the aforementioned conventional approaches. Specifically, longer-wavelength photo-irradiation corresponds to ultrabroadband ranges from sub-terahertz (sub-THz) to infrared (IR). Sub-THz–IR-irradiation first exhibits permeable views to various non-metallic materials^18,19^. Furthermore, transmission trends of these materials to sub-THz–IR-irradiation are variable per composition and wavelength^20,21^. Thus, ultrabroadband multi-wavelength optical measurements in sub-THz–IR bands identify non-metallic materials (e.g., polymer, glass, semiconductor, ceramic, and liquids) in a non-destructive manner^22,23^. Here, typical sub-THz–IR measurements contain spectroscopies at each band (THz time-domain spectroscopy: THz-TDS^24^, Fourier transform IR spectroscopy: FTIR^25^, and so on). Indeed, these spectroscopy techniques treat raw materials composing daily necessities and industrial products as fundamental evaluations at an early stage^26,27^.

However, these spectroscopies are still insufficient for the use of in-line pharma testing at manufacturing sites despite the above advantageous features. In such situations, bulky housing and sensitive configurations to external environmental conditions of existing spectroscopy equipment^28,29^ dominantly hinder their in-line setups. Here, the external environment includes humidity, indoor lighting, and so on. Furthermore, typical spectroscopies generally function as static and passive measurements by setting target samples within their bulky complicated equipment^30,31^. Inherently, industrial non-destructive testing techniques eagerly require treatments of products and infrastructure equipment in on-site situations. This concept pursues reliable non-destructive testing through comprehensive full evaluations of products at manufacturing sites and long-term consecutive monitoring of outdoor infrastructures during their operations throughout multiple weather and seasonal conditions.

Based on the above situation, non-destructive testing techniques should be completed without interfering with pristine environmental configurations (e.g., scale or speed) of targets in an on-site manner. Such ideas correspond to in-line testing of assembling flows at the pharmaceutical manufacturing sites. For addressing these fatal bottlenecks, the development of non-destructive in-line dynamic pharma inspection systems via ultrabroadband multi-wavelength sub-THz–IR monitoring is essential. Specifically, the above requirements contain unclosed, portable, and flexibly designable components for configurations of testing systems. In connection with these, several previous research treats portable spectroscopy techniques^32,33^. However, those narrow-band functions still hinder opportunities for wide-range non-destructive material composition identification. Therefore, efforts are crucially insufficient for developing non-destructive in-line dynamic pharma inspection systems via ultrabroadband multi-wavelength sub-THz–IR monitoring.

**Fig. 1:**
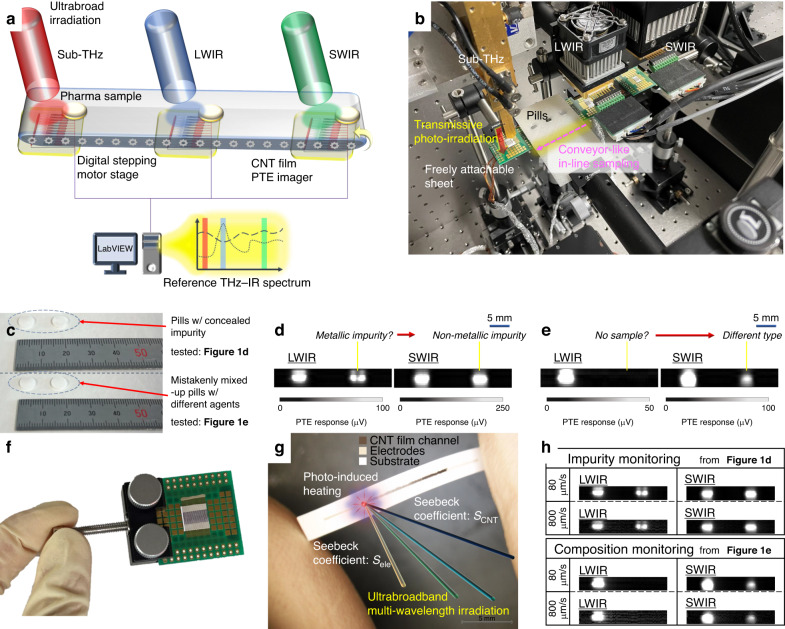
Conceptual diagram of this work. **a** Schematic diagram of the presented non-destructive in-line dynamic ultrabroadband sub-THz–IR pharma monitoring system with the CNT film PTE imager. LW/SW: longer-/shorter-wavelength. **b** Photograph of the experimental setup. Wavelength: 909 µm for sub-THz, 6.13 µm for LWIR, and 4.33 µm for SWIR. **c** Examples of defective pharma agent pills to be inspected by the presented device and system. For the top-half, this case study prepares two sedative pills. One on the right-hand side conceals a glass impurity in its centre position. For the bottom-half, this case study compares visibly indistinguishable sedative (left) and antipyretic (right) pills. **d** Impurity monitoring. **e** Material composition identification. **f** CNT film PTE imager. **g** PTE effect. **h** Higher-speed operations

To this end, this work demonstrates a non-destructive in-line dynamic inspection system for pharma agent pills with freely attachable functional imager sheets and the associated ultrabroad sub-THz–IR band multi-wavelength photo-monitoring (Fig. 1a). The above system develops in-line experimental setups by incorporating the thin-film sub-THz–IR imagers and compact multiple photo-sources at a single wavelength extracted in advance (Fig. 1b, extracted from reference spectroscopy-based databases as pharmaceutical-specific bands). This work inspects invisible detailed features (Fig. 1c) of concealed foreign substances (Fig. 1d) and pharma agents themselves (Fig. 1e) continuously in a non-contact and non-destructive manner with the in-line dynamic ultrabroadband sub-THz–IR monitoring system. Carbon nanotube (CNT) thin-film photo-thermoelectric (PTE) imager sheets, which play an essential role in the presented system, function in ultrabroadband regions over existing sensors in the comparable sensitivity with conventional narrowband detectors at each range, and facilitate multi-wavelength operations under external sub-THz–IR-irradiation (Fig. 1f,g). In these device setups, this work demonstrates higher-speed photo-monitoring operations of non-destructive in-line dynamic ultrabroadband pharma inspection by controlling material composition of CNT film PTE pixels (Fig. 1h). Finally, the presented device and system in this work handle pharma agent pills at the highest scan speed of 3 mm/s. Such approaches potentially detect concealed abnormalities of pharmaceuticals in an active live manner at manufacturing sites through comprehensive full evaluations before social distribution steps, over the existing inspection passively treating defective samples after being returned by users.

## Results

### Fundamental operation of the ultrabroadband imaging scanner

**Fig. 2:**
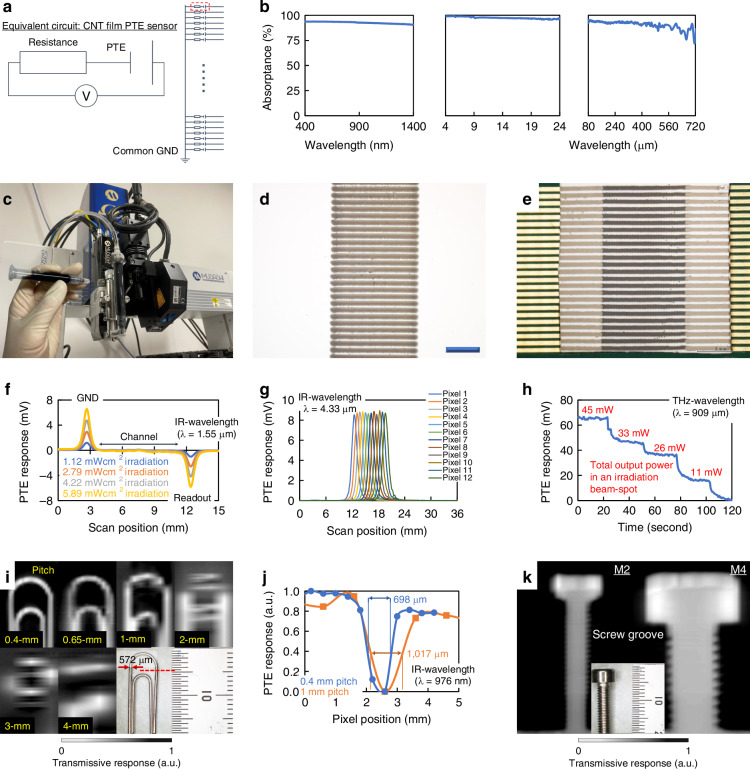
Fundamental operation of the ultrabroadband photo-imager. **a** Equivalent circuit of the imager. **b** Optical properties of the CNT film channel. **c** Air-jet dispenser for device printing. **d** Dispense-printed arrays of CNT film channels. **e** Photograph of the device. **f** Line-profiling of PTE response signals by scanning external photo-irradiation along a channel-length direction with the device pixel. **g** Distribution of PTE response signals with the device by scanning external photo-irradiation along a pixel-array direction. **h** Change in the PTE response signal of the device pixel with different intensities for external sub-THz-irradiation. **i** Quality change in transmissive 1D-scan IR PTE imaging of a metallic clip with the device per pixel array integration-density via dispense-printing (*λ*: 976 nm). **j** Full-width at half-maximum values in (i). **k** Transmissive 1D-scan IR PTE imaging of a metallic miniature screw by the device with a pixel array integration-density of 0.4 mm-pitch (*λ*: 976 nm)

In the presented non-destructive in-line dynamic pharma inspection system via ultrabroadband sub-THz–IR monitoring, functional photo-imager devices play an essential role. This work employs CNT films as pixel channel materials of the ultrabroadband PTE photo-imager. Figure 2a explains an equivalent circuit diagram of the CNT film PTE imager. Ohmic current-voltage characteristics of the device indicate it functions at zero-voltage-bias conditions (Fig. S1), and Fig. S2 introduces the fundamental PTE effect model. The CNT film PTE imager comprises parallel wiring integrations with each of the above pixels as one-dimensional (1D) array structures.

Figure 2b provides the fundamental optical properties of the CNT film channel. The CNT film exhibits highly efficient photo-absorptance values over 90% throughout ultrabroad MMW–Vis bands (see Spectroscopy in the Materials and methods section). In these measurements, this work subtracts reflectance values from primary obtained results via transmissive spectroscopy to evaluate photo-absorbing characteristics with CNT films. The device is suitable for operations in the presented non-destructive in-line dynamic pharma inspection system via ultrabroadband sub-THz–IR monitoring under these advantageous optical properties. For ultrabroadband efficient photo-absorption characteristics, the CNTs’ unique morphology and electronic nature dominantly play an essential role^34^. Under external MMW–Vis-irradiation, CNTs provide different photo-absorption mechanisms. In general, CNTs contain a wide range of diameters and lengths as their fundamental dimensions for handling forms of aqueous dispersions and network films. In mid-IR–Vis bands, respective CNTs with different diameters and the associated band gap energy levels exhibit specific peak wavelengths of external photo-irradiation. This situation is mainly because CNTs typically provide discretely quantised density of state as van Hove singularities, defining each band gap energy level, as directly linking from their diameters. Based on this theoretical background, CNT films comprising wide diameter distributions aggregate respective energy transition peak wavelengths (i.e., Interband shifting) into broadband photo-absorption characteristics in mid-IR–Vis regions. In MMW–far-IR bands, photo-irradiation triggers 1D axial plasmon resonances in CNTs (typically nm-diameters and µm-lengths) for the Drude absorption with delocalised free carriers within them (i.e., Intraband energy transition). Based on this theoretical background, CNT films exhibit respective 1D axial plasmon resonances and the associated specific energy transition peak wavelengths, aggregating them into broadband photo-absorption characteristics in MMW–far-IR regions with their wide length distributions. As this work employs unseparated aqueous CNT dispersions, where formed network films contain random distributions of diameters and lengths of each constituent tube, the presented PTE imager exhibits advantageous ultrabroadband efficient photo-absorption characteristics.

This work employs mechanical liquid printing techniques^35^ to fabricate the CNT film PTE imager. Figure 2c introduces the printing equipment (air-jet dispenser) and device materials in the form of liquid solution inks (e.g., aqueous CNT dispersions). The first process of the device fabrication flows is dispense-printing of CNT inks on water-absorbent membrane filter substrates (Fig. 2d). In addition to these photo-absorption channels, the CNT film PTE imager comprises electrodes for signal readout treatments under external photo-irradiation. As shown in Fig. S2, this channel-electrode junction functions as a photo-detection interface and is essential for device operations. In forming electrodes on the device, this work employs conductive pastes for dispense-printing. As respective constituent materials (photo-absorption channels and electrodes) are collectively solution-processable, the proposed fabrication process integrates each pixel of the CNT film PTE imager at high-yield (Fig. 2e) with the single dispense-printer via its mechanical spatial alignment. Carbon nanotubes and Device fabrication in the section “Materials and Methods” describe these detailed conditions.

Figure 2f provides line-profiling of PTE response signals along channel length directions for a pixel of the CNT film imager in scanning external photo-irradiation. The obtained result means that the device exhibits intensity peaks of photo-detection response signals at the heterogeneous-material interfaces in line with the PTE effect^36^. The respective CNT film channel-electrodes (GND and readout) interfaces offer reverse-polarity PTE response signals based on the above device operating mechanism^37^. The absolute values of PTE response signal intensities at these two photo-detection interfaces of the presented device are approximately the same. This situation means that the aforementioned air-jet mechanical dispense-printer sets channels and electrodes precisely in fabricating respective pixels of the CNT film PTE imager. This work utilises channel-GND junctions, providing positive-polarity PTE response signals under external irradiation in the above device structure, as a photo-detection interface of the imager.

Figure 2g compares PTE response signal intensities at multi-pixel photo-detection interfaces integrated as a 1D array imager device under external irradiation. Fig. S3 introduces detailed sensing systems for these measurements (Fig. 2f, g). In 12 pixels as an example, the presented device suppresses error ratios of PTE response signal intensities within 2.43% at photo-detection interfaces under external photo-irradiation. These results represent the significance of high-yield device fabrication techniques via mechanically alignable dispense-printing in this work. Photo-source and Signal readout in the section “Materials and methods” describe these detailed conditions.

In these photo-detection operations, PTE response signals of the CNT film imager are variable with changes in intensities of external irradiation (Fig. 2h, also see Fig. S4 for ultrabroadband operations). In these modulations, this work maintains external photo-irradiation to the CNT film PTE imager throughout the measurement and controls the intensity per constant time interval (e.g., 25 s each for Fig. 2h) during the single experiment. This situation is the reason why Fig. 2h and Fig. S4 employ time scales to their horizontal axes of respective graphs. This work converts the above change in PTE response signal intensities under external photo-irradiation to monochrome colour scales for applying the presented device as an image sensor. Fig. S5 further demonstrates that the CNT film PTE imager appropriately functions as a photo-sensor under external Vis-irradiation, in line with efficient absorptance values of the channel material reaching even the above bands.

Figure 2i presents an example of transmissive one-axis-scan photo-imaging of a metal clip (target) with the device under IR-irradiation. In the obtained PTE image, vertical and horizontal axes correspond to the respective scanning and pixel-alignment directions for the device. In a monochrome colour scale, the darker and blighter range refers to the higher and lower response signal intensities of each CNT film PTE pixel against transmissive photo-irradiation. The obtained PTE image clearly visualises the clip by detecting local reductions in transmissive response signal intensities of respective CNT pixels along a target shape due to reflection characteristics of the metal surface against external photo-irradiation. This is the reason the clip part looks blighter, and the rest appears darker by this IR scanning with the CNT film PTE imager. In general, denser pixel integrations of photo-imagers enable higher-spatial-resolution measurements^38^. This work designs the CNT film PTE imager in a 0.4 mm-pitch with the aforementioned device fabrication techniques via mechanically alignable air-jet dispense-printing (Fig. 2c–e). Figure 2j suppresses the image blurriness by 28% with the highest-density pixel array structure at present compared to a CNT film PTE device with conventional 1 mm-pitch sensor integrations via previous manual patterning methods^39^ under external IR-irradiation. Respective full-width at half-maximum values are 698 μm (0.4 mm pitch) and 1,017 μm (1 mm pitch) regarding a 572 μm width metal bar of the clip in the above demonstration. Furthermore, the presented device demonstrates higher-spatial-resolution measurements: visualising minute screw grooves (Fig. 2k) via one-axis-scan photo-imaging under external IR-irradiation. By incorporating these characteristics (operating regions, spatial pixel resolution, and sensitivity (Fig. S6)), the CNT film PTE imager plays an advantageous role against conventional photo-detectors^40–46^, and facilitates the presented non-destructive in-line dynamic pharma inspection in this work via ultrabroadband multi-wavelength sub-THz–IR monitoring (Table S1).

### Fundamental experimental setup for pharma monitoring

**Fig. 3:**
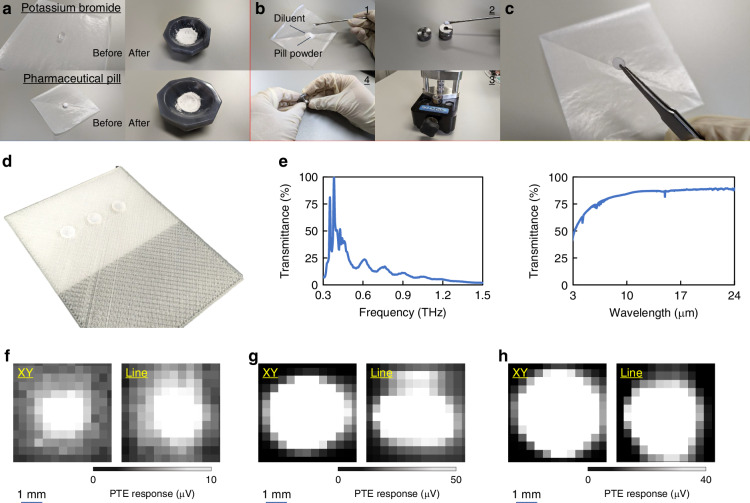
Preparation of pharma agent pills and their fundamental optical properties. **a**–**c** Pill formation process: powdering (**a**), compression (**b**), and a completed sample (**c**). **d** Experimental setup of pharma agent pills for imaging measurements. **e** Transmittance distribution of the normal sample (sedative) in sub-THz–IR bands. **f**–**h** Multi-wavelength imaging of the standard sample in sub-THz (f, *λ* - 909 μm), LWIR (g, *λ* - 6.13 μm), and SWIR (h, *λ* - 4.33 μm) bands with the device (Fig. 2e)

Following the above material and device configurations, a deeper understanding on preparations of pharma agent pills emphasises the advantageous usability of the presented non-destructive in-line dynamic ultrabroadband sub-THz–IR monitoring system in this work. This work again emphasises that the enriched material nature of the CNT film PTE imager and the associated high-yield device fabrication techniques (Fig. 2, Figs. S1, S2, S4–S6, Table S1, and Carbon nanotubes in the Materials and methods section) dominantly govern the fundamental performance of the following monitoring applications. Figure 3a–c introduces pill formation processes. This work configures standard conditions in forming pills as follows: 7-mm-diameter, 1-mm-thickness, and 5% concentration of pharmaceutical agents. Pharma agent pills in the section “Materials and Methods” describe detailed procedures. The presented non-destructive in-line monitoring system with CNT film PTE ultrabroadband sub-THz–IR imagers in this work carries pharma agent pills into the optical setup in a continuous and dynamic manner with a jig shown in Fig. 1b. Figure 3d and Fig. S7 introduce details of the setup for pharma agent pills (also see 3D printing in the Materials and methods section for tool preparations). Figure 3e exemplifies a mapping of optical transmittance values with a sedative pill in sub-THz–IR bands. The obtained result means that the pill sample exhibits transmittance values over 50% under external sub-THz–IR-irradiation. The above situation indicates that sub-THz–IR monitoring is effective for non-destructive pharma inspection. As the CNT film PTE imager is freely available for ultrabroadband monitoring on sub-THz–IR ranges as the above^47^, the presented non-destructive in-line dynamic pharma inspection system widely customises irradiation wavelength ranges of photo-sources beyond that of existing spectrometers, per optical property of targeted samples. Such configurations are essential for realising multi-wavelength photo-sensing as non-destructive material composition identification. The usability of the CNT film PTE imager (e.g., thin soft structures and freely attachable configurations) potentially installs the presented system into line manufacturing environments of pharma agent pills in an on-site manner without being bulky.

Figure 3f–h performs multi-wavelength photo-imaging of the standard sample by sub-THz (f), LWIR (g, LWIR: longer-wavelength IR), and SWIR (h, SWIR: shorter-wavelength IR) irradiation with the presented device-based in-line dynamic scanning under the above system. This work also provides results of two-dimensional (2D)-scan imaging with the sample as shown in the figure by employing the single pixel of the CNT film PTE device under the same three-wavelength external photo-irradiation. The obtained results emphasise the accuracy of the presented device and system, being comparable to that of 2D-scanning (higher-spatial-resolution, but longer measurement time^48^) for acquiring transmissive PTE images. Fig. S8 defines available size and concentration ranges of target pharma agent pills for the non-destructive in-line dynamic ultrabroadband monitoring system with CNT film PTE imagers. This work also confirms that the use of the CNT film PTE imager is principally applicable to handling real-scale pills as targets of photo-monitoring-based non-destructive inspection towards practical pharmaceutical applications (Fig. S9).

### Pharma monitoring demonstration

**Fig. 4:**
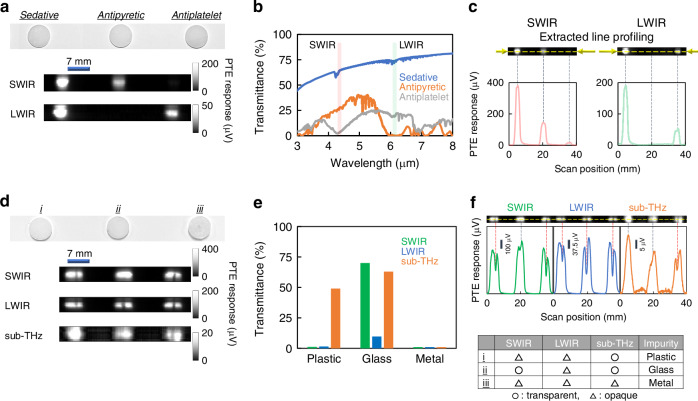
Non-destructive ultrabroadband and multi-wavelength optical inspection of defective pharma agent pills with the CNT film PTE imager. **a** Material composition identification of different agent samples. **b** Fundamental optical properties of inspection samples (in (**a**)). **c** Transmissive line-profiling from the obtained PTE image (in (**a**)). **d** Material composition identification of samples with different concealed foreign substances. **e** Fundamental optical properties of each foreign substance (in (**d**)). **f** Transmissive line-profiling from the obtained PTE image for identifying foreign substances (in (**d**))

Based on the above functional device and ultrabroadband system setting, the following demonstrations represent major advantages of sub-THz–IR-irradiation for non-destructive inspection of pharma agent pills. Figure 4a performs non-destructive material composition identification for different agent samples via broadband two-wavelength photo-monitoring with the CNT film PTE imager. Based on the conditions in Fig. 3, agents of the samples employed in Fig. 4 are as follows: sedative, antipyretic, and antiplatelet. The obtained results identify the material composition of the targeted pharma pills based on one-axis-scan transmissive LWIR and SWIR images by the device, which are visually indistinguishable. The sedative agent collectively indicates higher transmittance values to external photo-irradiation of two different wavelengths among the assessed samples. The antipyretic agent offers a selective transmission behaviour to SWIR-irradiation. The remaining antiplatelet agent provides a selective transmission trend to LWIR-irradiation.

These imaging results show a good agreement with the pre-obtained reference spectrum (Fig. 4b), which is the fundamental optical information of each inspected sample. This work handles various agents by grasping such spectrums of target samples as reference conditions in advance for non-destructive ultrabroadband pharma monitoring with CNT film PTE imagers. The reference spectrum extracts wavelengths with characteristic transmittance distributions from respective pharma agents, while CNT film PTE imagers sensitively perform photo-detection in ultrabroadband regions. The presented non-destructive ultrabroadband monitoring system completes its operations with experimental setups of compact photo-sources in the above wavelengths specific to targeted pharma agents, and detects abnormalities by classifying obtained response signal intensities for each sample with the device based on spectroscopy-driven characteristic transmittance values. This system freely switches photo-source setups per fundamental optical property of targeted agents, assisted by ultrabroadband photo-detection with CNT film PTE imagers.

Figure 4c provides line-profiling of transmissive response signal intensities of a device pixel from the obtained respective PTE images (Fig. 4a). The obtained results indicate that the device behaviours and brightnesses in respective images show a good agreement with the pre-obtained reference transmittances per each wavelength and sample. The superposition of the above two-wavelength transmissive images assures that targeted pills are non-metallic materials. For example, the centre sample is unconfirmable in LWIR imaging by the device. The use of only the LWIR image is insufficient to identify whether the centre sample is a metal object (reflective) or a photo-absorbent composition. A combination with the SWIR image reveals that the inspected target is a non-metallic material by visualising transmission features to the centre sample with the CNT film PTE imager.

The non-destructive ultrabroadband inspection system with CNT film PTE imagers in this work is also adaptable for monitoring hazardous foreign substances (i.e., impurity) concealed in pharma agents in addition to material composition identification of pills themselves. This work demonstrates non-destructive material composition identification of impurities themselves by adding higher-permeable sub-THz-irradiation within the presented device and system. Figure 4d performs transmissive ultrabroadband three-wavelength sub-THz–IR monitoring of pharma pills concealing different impurities with the CNT film PTE imager. Based on the conditions in Fig. 3, this experiment prepares three sedative agent pills as the main bodies of inspection targets for the above demonstration. The employed materials of impurities in Fig. 4d are as follows: plastic, glass, and metal. The obtained results identify respective impurities concealed in the pills based on one-axis-scan transmissive sub-THz, LWIR, and SWIR images by the device, which are visually indistinguishable. Despite the situation that the pills conceal the above three kinds of impurities, the combinations are unknown.

This work labels the pill samples as i, ii, and iii from the left-hand side to the right-hand side in Fig. 4d. Pill-i exhibits a vertical slit in the transmissive PTE image with external two-wavelength photo-irradiation of LWIR and SWIR bands. This vertical slit matches local reductions of transmissive photo-irradiation intensities in the pill from the correspondence between response signals of CNT film PTE pixels and the monochrome colour scale of the obtained image. The darker vertical slits in Fig. 4d correspond to impurities in the respective pills. The above situation indicates that two-wavelength photo-irradiation of LWIR and SWIR bands visualises the impurity in pill-i. The obtained result also visualises the impurity in pill-ii by transmissive LWIR imaging with the device. Finally, all three-wavelength photo-irradiation with the CNT film PTE imager in Fig. 4d collectively visualises the impurity in pill-iii. From another viewpoint, SWIR-irradiation is insufficient to distinguish the impurities among pill-i and pill-iii. Transmissive LWIR imaging with the device confuses the respective impurities in each pill. These tendencies, that the above visibilities of the employed impurities differ with photo-irradiation bands, properly reflect broad changes in transmittance values of various materials per composition and wavelength in sub-THz–IR regions. The above situation leads to non-destructive composition identification of concealed impurities in pharma agent pills by combining the pre-obtained reference optical database of materials and transmittance characteristics in sub-THz–IR bands (Fig. 4e). “Plastic”, one of the materials for impurities inside pills in Fig. 4d, provides lower transmittance values under external LWIR and SWIR irradiation than those in the employed sub-THz wavelength. “Glass” selectively offers a lower transmittance value to LWIR-irradiation. The remaining “Metal” does not exhibit transmission trends to the respective three-wavelength photo-irradiation via inherent reflective characteristics. Finally, the presented device and system in this work non-destructively identify the corresponding materials of impurities concealed in pharma agent pills as follows: i as plastic, ii as glass, and iii as metal (Fig. 4f, also see Fig. S10 for sample preparations).

Owing to huge expectations on sub-THz–IR measurements for the investigation field of material science, the fundamental optical databases of diverse compositions in the above bands are now available^49,50^, including Fig. 4e, via spectroscopy preparations in a static manner. Furthermore, the presented non-destructive ultrabroadband multi-wavelength sub-THz–IR monitoring system with CNT film PTE imagers potentially and conceptually brings such advantages in on-site dynamic configurations of pharmaceutical inspection applications.

### In-line real-time pharma monitoring

**Fig. 5:**
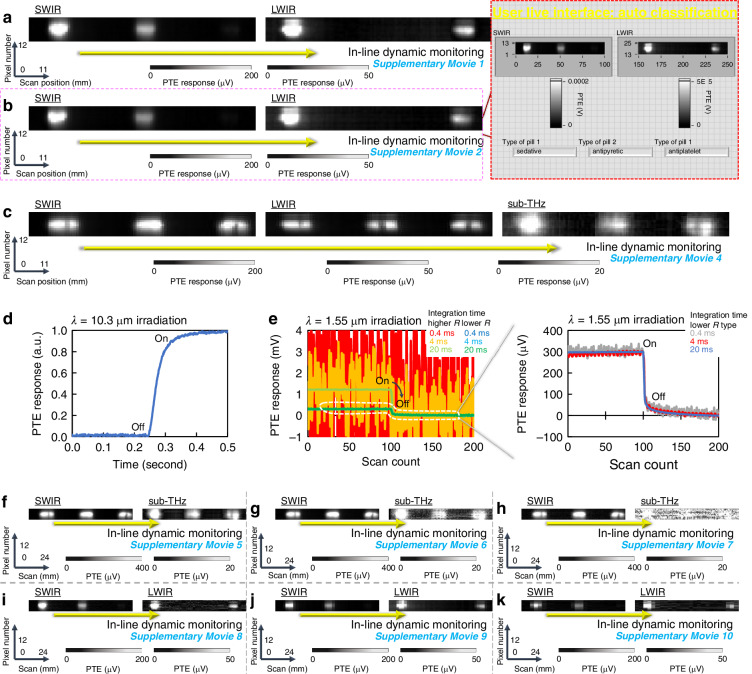
Non-destructive in-line and dynamic ultrabroadband inspection of defective pharma agent pills with the CNT film PTE imager. **a** In-line material composition identification (same samples as Fig. 4a). **b** Automatic classification of pharma agent types with a transmittance database-driven manner. **c** In-line foreign substance identification (same defects as Fig. 4d). **d** Transient response of the CNT film PTE pixel to external photo-irradiation. **e** Change in behaviours of the device with CNT compositions in higher-speed operations. **f**–**h** Changes in in-line image quality with readout speed of response signals from the device at a fixed spatial scanning resolution (100 μm/scan-step): 0.70 s/scan-step (**f**), 0.50 s/scan-step (**g**), and 0.45 s/scan-step (**h**). **i**–**k** Changes in in-line image quality with spatial scanning resolutions at a fixed readout speed (0.5 s/scan-step): 0.4 mm/scan-step (**i**), 1 mm/scan-step (**j**), and 1.5 mm/scan-step (**k**)

By incorporating the above findings and techniques, this work demonstrates non-destructive in-line dynamic material composition identification for defective pharma agent pills via ultrabroadband multi-wavelength sub-THz–IR monitoring with CNT film PTE imagers. Figure 5a first handles the same samples as Fig. 4a. Supplementary Movie 1 (10x speed) introduces results of the above experiment. The obtained results indicate that the presented device and system in this work properly function in an in-line dynamic setup as non-destructive pharma inspection. The above setup aligns each optical system (compact photo-sources and freely attachable CNT thin-film PTE imager sheets) to the same single line. Pharma pills move continuously among photo-sources and devices along the system alignment line (perpendicular to optical paths), while the presented device and system provide non-destructive ultrabroadband multi-wavelength transmissive images in real-time.

Figure 5b then introduces automatic classification of pharma agent types by applying the presented device and system with a transmittance database-driven manner. This system links the above real-time non-destructive in-line dynamic broadband multi-wavelength views from CNT film imagers to the pre-obtained fundamental transmittance values of each pharma agent pill. The above application sets the fundamental transmittance values of respective samples as threshold conditions linked to the agent name, and real-time PTE response signal intensities of the device automatically identify materials of targeted in-line pills. Supplementary Movie 2 (10x speed) presents the live video of Fig. 5b. Such automatic classification functionality potentially enriches the universality of non-destructive in-line dynamic pharma monitoring by developing a broadband database of the fundamental optical characteristics for possible impurity materials (Fig. S11) along with wide-range photo-detection with the CNT film PTE imager.

While the above demonstration develops an early-stage database across far-IR–Vis regions, similar efforts on broad THz bands play a key role in the next scope of this work to perform further efficient non-destructive pharma monitoring owing to the inherent fingerprint spectrum of diverse constituent polymer compositions of pills. As THz-based static spectroscopy has gradually emphasised its presence as an analysis tool of pharma agents at drug discovery stages with thinly sliced samples^51^ prior to manufacturing phases, the experimentally demonstrated concept in this work provides a potential to integrate such detailed classification of constituent chemical foundations themselves into in-line dynamic setups. Fig. S12 demonstrates non-destructive in-line dynamic exception composition identification of pharma agent pills (also see Supplementary Movie 3 (10x speed)) with the same mechanism as Fig. 5b via broadband multi-wavelength photo-monitoring by CNT film PTE imagers. These PoCs emphasise the potential for adapting the presented device and system in this work at practical pharmaceutical manufacturing sites.

Figure 5c performs non-destructive in-line dynamic impurity identification concealed in pharma agent pills (same defects as Fig. 4d) under transmissive ultrabroadband multi-wavelength sub-THz–IR monitoring with CNT film PTE imagers. Supplementary Movie 4 (10x speed) introduces results of the above experiment. The obtained results indicate that the presented device and system in this work also properly function as non-destructive in-line dynamic inspection techniques in ultrabroadband photo-monitoring containing even sub-THz-irradiation.

Following the above fundamental performances, this work demonstrates higher-speed non-destructive system operations of in-line dynamic broadband multi-wavelength pharma monitoring with controlling material composition of CNT film PTE imagers. Figure 5d presents a transient waveform of photo-induced response signal intensities with the CNT film PTE pixel. These electrical signal readouts require shorter integration time for higher-speed device operations. Device operations with shorter integration time typically induce more significant noise signals^52^. This work inhibits noise signals in higher-speed signal readout operations for photo-detection by adopting a lower electrical resistance CNT film channel for the PTE pixel material. Figure 5e introduces transient waveforms of photo-induced response signal intensities for different compositions of CNT film PTE pixels. This work evaluates two types of CNT film channels with three different integration time; respective electrical resistance values are 10.8 kΩ (channel 1, surfactant ratio: 3 wt%) and 660 Ω (channel 2, surfactant ratio: 0.4 wt%). The obtained result exhibits crucial increases in noise signals of the CNT film PTE sensor (channel 1) during photo-detection under higher-speed signal readout operations faster than 4 milliseconds (ms). The CNT film PTE sensor (channel 2) provides clear transient waveforms to external photo-irradiation under the above signal readout speed. This measurement sets theoretical signal readout speed to 20 ms, 4 ms, and 0.4 ms by the equipment configuration. The number of power line cycle (NPLC) parameters define the above three signal readout speed conditions (NPLC 1: 20 ms, NPLC 0.2: 4 ms, and NPLC 0.02: 0.4 ms). This work conducts optical measurements under the single-pixel signal readout speed: theoretically 20 ms so far.

The right-hand side graph of Fig. 5e extracts waveforms of the CNT film PTE sensor (channel 2) from the left-hand-side one. In the signal readout at 20 ms speed, the Seebeck coefficient of each CNT film controls photo-response intensities of the PTE sensor (channels 1 and 2). The Seebeck coefficients are as follows: 55 μV/K (channel 1)^48^ and 30 μV/K (channel 2). In general, Johnson-Nyquist signals (proportional to square root values of the electrical resistance) dominantly account for noise behaviours in operations of PTE devices^53^. The employed two kinds of CNT film channels in this work are collectively semiconducting-metallic-unseparated types. This work considers that surfactant ratios in respective aqueous CNT dispersions potentially degrade electrical resistances of subsequent films as insulating residues. Figures S13–S20 summarisethese material composition strategies for the CNT film PTE imager towards faster operations of the non-destructive photo-monitoring system.

Figure 5f–h evaluates dynamical operation speed of the non-destructive in-line pharma inspection system via ultrabroadband multi-wavelength photo-monitoring with the CNT film PTE imager (channel 2) adapting to faster signal readout conditions. This work performs impurity identification (same setup as Fig. 5c) concealed in pharma agent pills. Specific experimental conditions are as follows: 0.70 s/scan-step (f: theoretical single pixel readout speed of 20 ms), 0.50 s/scan-step (g: theoretical single pixel readout speed of 4 ms), and 0.45 s/scan-step (h: theoretical single pixel readout speed of 0.4 ms) at a fixed spatial scanning resolution of 100 μm with the presented device and system. The obtained results experimentally emphasise that the use of lower electrical resistance CNT film PTE imagers enables operations of the entire non-destructive in-line dynamic ultrabroadband pharma monitoring system at the fastest speed of 0.50 s/scan-step. The higher-intensity IR irradiation (SWIR in the figure) compared to the employed sub-THz band further allows the system operation at 0.45 s/scan-step. Supplementary Movie 5–7 (5: 0.70 s/scan-step, 6: 0.50 s/scan-step, and 7: 0.45 s/scan-step (10x speed)) provides live videos of Fig. 5f–h.

In addition to the above controls of readout speed for CNT film ultrabroadband PTE imagers, this work also evaluates spatial scanning resolutions of the non-destructive in-line dynamic pharma monitoring system. The resolution conditions of performed experiments in this work are in a range of 0.1–0.4 mm/scan-step so far. The total operation speed (mm/s) of the non-destructive in-line dynamic pharma monitoring system with CNT film ultrabroadband PTE imagers divides spatial scanning resolutions (mm/scan-step) by the above signal readout speed. Figure 5i–k demonstrates material composition identification of pharma agent pills (same setup as Fig. 5a) as controls of spatial scanning resolutions and the associated total system operation speed. Specific experimental conditions are as follows: 0.4 mm/scan-step (Fig. 5i, converted into 0.8 mm/s), 1 mm/scan-step (Fig. 5j, converted into 2 mm/s), and 1.5 mm/scan-step (Fig. 5k, converted into 3 mm/s) at a fixed readout speed of 0.50 s/scan-step with the presented device and system. The obtained results experimentally emphasise that the non-destructive in-line dynamic broadband pharma monitoring system in this work correctly visualises shapes and optical properties of respective targeted pills at the highest speed of 2 mm/s (still providing the appropriate imaging information at 3 mm/s). Supplementary Movie 8–10 (8: 0.8 mm/s, 9: 2 mm/s, and 10: 3 mm/s (10x speed)) provides live videos of Fig. 5i–k. By incorporating these findings and techniques, this work demonstrates non-destructive dynamic broadband multi-wavelength photo-monitoring of pharma agent pills in consecutive in-line sample-delivery processes mimicking their manufacturing sites, while clarifying that material designs of CNT film PTE imagers and equipment configurations dominantly govern system operation speed towards practical social implementation.

### Outlook for system automation

As this work performs non-destructive in-line dynamic pharma monitoring with CNT film PTE imagers under external broadband sub-THz–IR-irradiation in transmissive optical systems so far, hybrid system setups with reflective scanning further provide detailed surface status of metal impurities concealed in pills (Fig. S21). At this moment, the presented device and system automate their operations within a scanning length range of the optical stepping motor stage (500 mm) via synchronising it with the datalogger (readout: response signals from CNT film PTE imagers) by LabVIEW programmes. Under the pill setting condition of 7-mm-diameter and 10-mm-interval (along with experimental setups for the presented measurements in this work), the current configurations theoretically handle up to 30 pills per single in-line scanning. As an optical stepping motor stage with a scanning length range of 800 mm is also commercially available as lab-scale equipment, the above maximum number of pills in in-line monitoring potentially reaches 47. By assuming the use of customised optical stepping motor stages with longer scanning ranges, the CNT film PTE imager-based in-line broadband sub-THz–IR monitoring system theoretically handles 635 pills per hour with the presented fastest imaging speed of 3 mm/s. Based on these discussions, one of the essential next scopes from this work is expanding the experimental setup of the presented device and system beyond the lab-scale to assess the applicability of the demonstrated non-destructive automatic classification (Supplementary Movie 2) towards practical in-line pharma monitoring at dynamic manufacturing sites.

## Discussion

The above experimental demonstration emphasises the advantageous usability of the presented device and system in this work among typical and fundamental pharmaceutical testing methods^9,10,26,27,32,33,54^, as summarised in Table 1 for their features. Representative pharma testing methods are as follows: spectroscopy, pulsed laser imaging, probing, and Vis cameras. Spectroscopies visualise constituent functional groups (IR) or ingredients (THz) by bringing pill samples into the equipment and overlaying fingerprint spectrum cues in detail. From the viewpoint of simple operations, the probe unit directly observes designated target areas and enables compact evaluations at a single spot with narrow IR band measurements. The Vis camera effectively facilitates detailed status sensing and edge-detection type crack monitoring of pill surfaces by machine learning-driven data processing.

**Table 1:** Benchmarking chart of the fundamental performances and specifications among the representative pharma monitoring systems based on photo-sensing techniques

	Operating band	Operating speed	System mechanism	Operating style	Available pill size	Available pill concentration	Analysing targets
This work	sub-THz–IR (MMW–Vis for imagers)	3 mm/s (imaging)	Ultrabroad & multi-wavelength array-imaging	In-line & dynamic @ indoor sites (ubiquitous)	up to 7-mm-thickness	up to 20 wt%	Indistinguish-able agents, Classification :inner hidden opaque impurities
THz-TDS^26^	THz	over several hours (imaging)	Spectroscopy	Samples in equipment (static way) @ closed & dehumidify	up to 0.5-mm- thickness	up to pristine types	Indistinguish-able agents
FTIR^27^	MIR	N.A. (single point)	Spectroscopy	Samples in equipment (static way) @ closed & dehumidify	N.A.	over 10 wt%	Indistinguish-able agents
Laser^54^	THz	over several hours (imaging)	Single-band *xy*-sensing	Samples in equipment (static way) @ closed & dehumidify	up to 2-mm thickness	10–30 wt%	Coating films
Surface colour^9^	UV	30 mm/s (imaging)	Hyper-spectral	In-line & dynamic @ shielded containers	up to 500-µm- thickness	N.A.	Surface area
Machine learning^10^	Vis	N.A. (camera)	Edge-detection	Samples on static stages w/ cameras	N.A.	N.A.	Deformations
Probe^32^	MIR	1 sample per sec. (single- spot)	ATR	In-line & dynamic @ designated containers	N.A.	up to 23 wt%	Slurries
In-line^33^	UV	1 sample per 50 ms (camera)	Hyper-spectral	In-line & dynamic @ shielded containers	N.A.	up to 3 wt%	Surface density

Among them, the presented system functions while maintaining fundamental monitoring performances comparable with existing methods under a compact in-line dynamic experimental environment. Simultaneously, material composition identification of concealed impurities by ultrabroadband photo-monitoring containing even sub-THz-irradiation in the system, assisted by enriched properties of CNT film PTE imagers, brings the additional potential to non-destructive pharma testing techniques. While the presented device and system play a complementary role with static spectroscopy techniques (e.g., THz-TDS), the former properly exhibits a comparable sensitivity with the latter by handling pharma agent pills at thicknesses of hundreds of µm–a few mm in a non-destructive in-line dynamic monitoring manner. This work calculates the specific operation sensitivity of the in-line monitoring system with CNT film imagers by providing error ratios of transmittance values in handling pharma pills within 5% (Fig. 4b, c: “antipyretic (42.43% by spectroscopy/37.86% by PTE)” and “antiplatelet (25.75% by spectroscopy / 28.07% by PTE)” calibrated by “sedative”). In this accurate pharma monitoring with the presented system, the CNT film PTE imager dominantly governs such performances by offering ultrabroad operation bands over those of typical optical devices under the comparable photo-detection sensitivity with that of respective narrow wavelength-range sensors (e.g., THz or IR types) in each region (Fig. S6).

Following these, further related efforts potentially enrich the functions of the presented device and system in this work. One specific example is implementing reflective units into the current transmissive optical system for non-destructive in-line dynamic ultrabroadband photo-monitoring with CNT film PTE imagers, which treats higher concentrations of pharma agent pills inspired by the typical attenuated total reflection (ATR) methods^25,55^. From another viewpoint, optimising readout circuits anticipates faster pharma inspection at several hundreds Hz scanning corresponding to the fundamental performance of imagers (e.g., one potential targeted value is 200 mm/s^56^, and a potential operation speed of the presented system anticipates 270 mm/s by assuming the equipment functions along the time constant value of CNT film PTE pixels^57–59^). Following these outlooks, device fabrication techniques also play an essential role in further performance enhancement of the presented non-destructive in-line dynamic broadband sub-THz–IR pharma monitoring system. Fig. S22 indeed demonstrates that the mechanical transition from the current air-jet dispensing to electric field-driven inkjet-printing potentially brings high spatial resolutions to the CNT film PTE imager at a range of pixel integration pitches within 10 µm.

In conclusion, the CNT film PTE imager-based ultrabroadband in-line dynamic system non-destructively identifies ingredient materials and impurity compositions of pharma agent pills under the following conditions: in the mobile desktop setup, throughout 4.33–909 μm wavelengths, and at the fastest scanning speed of 3 mm/s. Specifically, this system treats consecutively flowing pills at the largest thickness of 7 mm and the highest agent concentration of 20% (Fig. S8). Towards the targeted performance of 200 mm/s for practical social implementation, the theoretically estimated operation speed of the presented system reaches 270 mm/s (3 mm/s at this moment) by optimising readout circuits for CNT film PTE imagers (fastest time constant: 5 ms), where the employed datalogger in this work still requires 0.45 s per scan-step.

In demonstrating these non-destructive pharma monitoring techniques, two properties based on CNT film channels dominantly govern the fundamental device and system operations from the viewpoint of material science. One is ultrabroadband photo-absorption characteristics by CNT films, and the remaining is their solution-processable configurations. Based on the former characteristics, operators complete the system development for pharma monitoring by employing CNT film PTE imagers against ultrabroadband multi-wavelength photo-irradiation without customising respective detector devices of different mechanisms, advantageously simplifying experimental preparation processes. The latter situation further facilitates cost-effective uncooled air-exposed liquid printing device fabrication of photo-imagers over thermal evaporation or sputtering and so on (larger amount of material consumption for covering entire areas with masks) by selectively patterning CNT inks via mechanically controlled spatial alignment. The liquid-printing-based device fabrication especially handles diverse thin-film supporting materials by completing respective processes without intensive vacuuming or high-temperature heating, which potentially induces crucial mechanical deformations of such sheet substrates. This configuration is also essential in designing freely attachable thin-film CNT PTE imager sheets regardless of implementation environments, such as narrow and intricate belt conveyor-based assembling lines of pharmaceutical products. While several materials exhibit promising potentials for functional optical devices (e.g., gallium arsenide, graphene, conductive polymers, vanadium oxide, transition metal dichalcogenides, and so on)^43–45,60,61^, the use of CNTs plays a leading role in collectively satisfying informative ultrabroadband efficient photo-absorption characteristics and easy-to-handle solution-processable configurations for developing in-line pharma monitoring photo-imagers.

From the viewpoint of typical industrial inspection, the use of X-ray scanning significantly facilitates non-destructive inner visualisation of invisible opaque parts with ultrahigh spatial resolution (e.g., tens of µm). While X-ray techniques inevitably require professional care of the damage on targets, not only themselves but also whole experimental sites (e.g., shielding room) to avoid consecutively reported lab-scale accidents involving human operators of testing systems over decades^62^, such configurations regulate their use to well-trained experts. For extracting information, X-ray techniques selectively specialise in the non-destructive visualisation of metallic parts from invisible opaque containers, even from deep spots.

Based on these situations, the presented sub-THz–IR monitoring in this work plays a complementary role in non-destructive pharmaceutical quality testing applications. Sub-THz–IR-irradiation first exhibits similar non-destructive optical permeability to diverse opaque materials with X-ray techniques, and advantageously provides opportunities for non-invasive inner inspection to broader operators owing to the lower photon energy of the former than the latter (e.g., a few meV via THz, while thermal-radiation at room temperature reaches 26 meV). Such perfectly non-destructive inner inspection under sub-THz–IR-irradiation further enriches conventional testing methods by composition identification among diverse non-metallic materials, being typically and collectively passed over in X-ray techniques due to their intensive permeability, unique to longer-wavelength photo-monitoring. This selective nature is mainly because that sub-THz–IR-irradiation inherently provides different transmittance values per wavelength and scanned composition, and the associated aggregation of their changes via broadband photo-monitoring leads to advantageous identification of non-metallic materials (e.g., ceramic? wood? semiconductor? plastic? and so on). While industrial products mostly comprise non-metallic materials (e.g., pharmaceutical pills: polymer agent and plastic coating), composition identification of them via sub-THz–IR-irradiation essentially contributes to non-destructive inspection together with ultra-high resolution inner visualisation of metallic parts by X-ray techniques. Under such discussions, the complementary use of sub-THz–IR-irradiation and X-ray techniques potentially induces synergetic effects in non-destructive pharma inspection from the initial in-line dynamic screening at manufacturing sites with the former to static detailed visualisation to pre-extracted pills from assembling lines by the latter.

## Materials and methods

### Spectroscopy

This work conducted UV-Vis-NIR spectroscopy measurements (UV-2600i, ISR-2600Plus, Shimazu Co.) to evaluate the fundamental optical properties of CNT films, which were the channel material of employed PTE imagers. The scan range and resolution were 220–1400 nm and 1 nm. For analysing the fundamental features of pharma agent pills, this work also performed Fourier Transform Infrared spectroscopy measurements (FTIR: IRTracer-100, Shimadzu Co.) The scan range and resolution were 7800–350 cm^−1^ and 0.5 cm^−1^. In Fig. 2b, this work further included THz time-domain spectroscopy measurements (THz-TDS: TAS7x00TS, Advantest Co.), in addition to the above UV-Vis-NIR and FTIR evaluations.

### Carbon nanotubes

This work employed aqueous single-walled CNT dispersions of semiconducting-metallic-unseparated (ZEONANO SG101 (Zeon Co., 0.5 wt%) and EC-DH (Meijo Nano Carbon Co., 0.2 wt%)) types. The CNT films have non-oriented structures.

### Device fabrication

This work conducted printing of the CNT film PTE imager with a non-contact air-jet mechanical auto dispenser. The experimental setups are as follows: a desk-top robot (SHOTMASTER®300ΩX SM300OMEGAX-3A-SS, Musashi Engineering, Inc.), a control unit (Hyper Jet®2 MJET-H-2-CTR3, Musashi Engineering, Inc.), a temperature controller (TCU-02-MU, Musashi Engineering, Inc.), a software for robot controlling (350PC-SMART, Musashi Engineering, Inc.), and an application for pattern editing (MuCADV, Musashi Engineering, Inc.). The printable device fabrication includes the following processes: patterning arrays of CNT inks on 70 µm-thick membrane filters (supporting substrate material: 200 nm pore, C020A025A, Advantec Ltd.) and wiring electrodes at both ends of film channels by dispensing a silver particle-binder resin-mixed conductive paste (ELEPASTE NP1, TAIYO INK MFG Co. Ltd.). This work then placed the dispense-printed sheet on a circuit board (SOP IC conversion board, SSP-41, Sunhayato Corp.) and manually made electrical bonding with the above conducting paste. The curing temperature condition was set at 120 °C for 10 min.

### Photo-source

This work employed three types of photo-sources for optical measurements: quantum cascade lasers (*λ* = 4.33 µm QCL L12004-2310 H-E, *λ* = 6.13 µm QCL L12006-1631H-E, Hamamatsu Photonics K.K.) in the SWIR and LWIR bands, and a frequency multiplier (*λ* = 909 µm 320–340 GHz, Virginia Diodes, Inc.) in the sub-THz region. This work also utilised three wavelength photo-irradiation for the fundamental device evaluations: a beam-expanded semiconducting fibre diode (*λ* = 976 nm, GBE05-B, BL976-PAG900, Thorlabs, Inc.), a semiconducting fibre diode (*λ* = 1.55 μm, FPL1009S, Thorlabs, Inc.), and a CO_2_ gas laser (*λ* = 10.3 μm, L4, Access Laser Co.). Respective photo-sources employed in this work cost a price range of 2 k$–14 k$, while that of static spectrometers or X-ray modules sometimes reaches beyond 100 k$. As the presented CNT film PTE imager-based in-line pharma monitoring system requires widely commercialised stages or datalogger circuits except for photo-sources, the demonstrated concept in this work provides a potential for large-scale non-destructive applications.

### Signal readout

This work employed a multiplexer datalogger (34933A, KEYSIGHT TECHNOLOGIES Inc.) to read out PTE response signals of the device. This datalogger linked ends of multiple pixels (readout electrode and ground). This work also utilised a motorised digital stepping stage (Motorized Stage, Sigma Koki Co.) and its controller (SHOT-304GS, Sigma Koki Co.) for spatial scanning measurements. This stage moved a step each time the datalogger read out one count of response signals from the CNT film PTE imager.

### Pharma agent pills

To demonstrate pharmaceutical identifications, this work employed Antipyretic (Ethenzamide ”Yoshida”, Yoshida Pharmaceutical K.K.), Antiplatelet (Ticlopidine Hydrochloride Tablets 100 mg “Sawai”, Sawai Pharmaceutical K.K.), and potassium bromide (KBr Chunks 100 g, S. T. Japan Inc) agents. KBr played sedative and diluent roles. This work also used a tablet pressure moulding machine (Pixie mini hydraulic press, 181–1400, S. T. Japan Inc) with a 7 mm diameter die set (161–1010, S. T. Japan Inc). These instruments apply mechanical pressurise up to 2.5 tonnes.

### 3D printing

For fabricating the pharma agent pill folder, this work conducted 3D resin printing (Value 3D Magix MF-2500 EP II, MUTOH INDUSTRIES Ltd.) with a CAD software (AUTODESK TINKERCAD). The type of resin was polylactic acid. The minimum processing resolution was 50 µm in *xy* and 100 µm in *z* directions.

## Supplementary information

Supplementary Information

Supplementary Movie 1_Real-time video for Figure 5a (10x speed)

Supplementary Movie 2_Real-time video for Figure 5b (10x speed)

Supplementary Movie 3_Real-time video for Supplementary Figure 12 (10x speed)

Supplementary Movie 4_Real-time video for Figure 5c (10x speed)

Supplementary Movie 5_Real-time video for Figure 5f (10x speed)

Supplementary Movie 6_Real-time video for Figure 5g (10x speed)

Supplementary Movie 7_Real-time video for Figure 5h (10x speed)

Supplementary Movie 8_Real-time video for Figure 5i (10x speed)

Supplementary Movie 9_Real-time video for Figure 5j (10x speed)

Supplementary Movie 10_Real-time video for Figure 5k (10x speed)

## Data Availability

The data that support the figures and other findings in this paper are available from the corresponding authors upon reasonable request.

## References

[1] Grangeia, H. B. et al. Quality by design in pharmaceutical manufacturing: a systematic review of current status, challenges and future perspectives. *Eur. J. Pharmaceutics Biopharmaceutics* **147**, 19–37 (2020). 10.1016/j.ejpb.2019.12.007 31862299

[2] Hauk, C., Hagen, N. & Heide, L. Identification of substandard and falsified medicines: influence of different tolerance limits and use of authenticity inquiries. *Am. J. Tropical Med. Hyg.* **104**, 1936–1945 (2021). 10.4269/ajtmh.20-1612 PMC8103440 33788775

[3] Arden, N. S. et al. Industry 4.0 for pharmaceutical manufacturing: preparing for the smart factories of the future. *Int. J. Pharmaceutics* **602**, 120554 (2021). 10.1016/j.ijpharm.2021.120554 33794326

[4] Ghosh, R. et al. Automation opportunities in pharmacovigilance: an industry survey. *Pharm. Med.* **34**, 7–18 (2020). 10.1007/s40290-019-00320-0 32036574

[5] Rani, N. S., Nithusha, V. K. & Roshna, T. P. Automatic recognition and verification of defective tablet blisters using entropy based filtering and histogram processing. *Int. J. Appl. Eng. Res.* **10**, 13155–13167 (2015).

[6] Derganc, J. et al. Real-time automated visual inspection of color tablets in pharmaceutical blisters. *Real.-Time Imaging* **9**, 113–124 (2003).

[7] Hole, G., Hole, A. S. & McFalone-Shaw, I. Digitalization in pharmaceutical industry: what to focus on under the digital implementation process? *Int. J. Pharmaceutics: X* **3**, 100095 (2021). 10.1016/j.ijpx.2021.100095 PMC8528719 34712948

[8] Watters, J. K. & Biernacki, P. Targeted sampling: options for the study of hidden populations. *Soc. Probl.* **36**, 416–430 (1989).

[9] Al Ktash, M. et al. Characterization of pharmaceutical tablets using UV hyperspectral imaging as a rapid in-line analysis tool. *Sensors* **21**, 4436 (2021). 34203526 10.3390/s21134436 PMC8271527

[10] Holtkötter, J. et al. Development and validation of a digital image processing-based pill detection tool for an oral medication self-monitoring system. *Sensors* **22**, 2958 (2022). 35458941 10.3390/s22082958 PMC9028233

[11] Udayakumar, G. P. et al. Biopolymers and composites: Properties, characterization and their applications in food, medical and pharmaceutical industries. *J. Environ. Chem. Eng.* **9**, 105322 (2021).

[12] Seo, K. S. et al. Pharmaceutical application of tablet film coating. *Pharmaceutics* **12**, 853 (2020). 32911720 10.3390/pharmaceutics12090853 PMC7558083

[13] Kim, S. S. et al. Review of semiconductor flash memory devices for material and process issues. *Adv. Mater.* **35**, 2200659 (2023). 10.1002/adma.202200659 35305277

[14] Javaid, M. et al. Sensors for daily life: a review. *Sens. Int.* **2**, 100121 (2021).

[15] Tonouchi, M. Cutting-edge terahertz technology. *Nat. Photonics* **1**, 97–105 (2007).

[16] Rogalski, A. History of infrared detectors. *Opto-Electron. Rev.* **20**, 279–308 (2012).

[17] Li, K. et al. Stretchable broadband photo-sensor sheets for nonsampling, source-free, and label-free chemical monitoring by simple deformable wrapping. *Sci. Adv.* **8**, eabm4349 (2022). 35544563 10.1126/sciadv.abm4349 PMC9094654

[18] Goryanin, I. et al. Passive microwave radiometry in biomedical studies. *Drug Discov. Today* **25**, 757–763 (2020). 32004473 10.1016/j.drudis.2020.01.016

[19] Valušis, G. et al. Roadmap of terahertz imaging 2021. *Sensors* **21**, 4092 (2021). 34198603 10.3390/s21124092 PMC8232131

[20] Martin, M. et al. Infrared thermography in the built environment: a multi-scale review. *Renew. Sustain. Energy Rev.* **165**, 112540 (2022).

[21] Koch, M. et al. Terahertz time-domain spectroscopy. *Nat. Rev. Methods Prim.* **3**, 48 (2023).

[22] Jiang, Y. Y., Ge, H. Y. & Zhang, Y. Quantitative analysis of wheat maltose by combined terahertz spectroscopy and imaging based on Boosting ensemble learning. *Food Chem.* **307**, 125533 (2020). 31634763 10.1016/j.foodchem.2019.125533

[23] Ma, J. et al. Comparison of spectral properties of three hyperspectral imaging (HSI) sensors in evaluating main chemical compositions of cured pork. *J. Food Eng.* **261**, 100–108 (2019).

[24] Baydin, A. et al. Time-domain terahertz spectroscopy in high magnetic fields. *Front. Optoelectron.* **14**, 110–129 (2021). 36637783 10.1007/s12200-020-1101-4 PMC9743882

[25] Tiernan, H., Byrne, B. & Kazarian, S. G. ATR-FTIR spectroscopy and spectroscopic imaging for the analysis of biopharmaceuticals. *Spectrochim. Acta A: Mol. Biomol. Spectrosc.* **241**, 118636 (2020). 32610215 10.1016/j.saa.2020.118636 PMC7308041

[26] Suzuki, D. & Kawano, Y. Terahertz imaging and spectroscopy as a tool for non-destructive and non-contact quality inspections of medical drugs and polymer films. *Bunseki Kagaku* **66**, 893–899 (2017).

[27] McCrae, K. et al. Assessing the limit of detection of Fourier-transform infrared spectroscopy and immunoassay strips for fentanyl in a real-world setting. *Drug Alcohol Rev.* **39**, 98–102 (2020). 31746056 10.1111/dar.13004

[28] Guerrero-Pérez, M. O. & Patience, G. S. Experimental methods in chemical engineering: Fourier transform infrared spectroscopy—FTIR. *Can. J. Chem. Eng.* **98**, 25–33 (2020).

[29] Puc, U. et al. Ultra-broadband and high-dynamic-range THz time-domain spectroscopy system based on organic crystal emitter and detector in transmission and reflection geometry. *Adv. Photonics Res.* **2**, 2000098 (2021).

[30] Ulatowski, A. M., Herz, L. M. & Johnston, M. B. Terahertz conductivity analysis for highly doped thin-film semiconductors. *J. Infrared, Millim., Terahertz Waves* **41**, 1431–1449 (2020).

[31] Kafle, B. et al. A portable dry film FTIR instrument for industrial food and bioprocess applications. *Anal. Methods* **16**, 4310–4321 (2024). 38888190 10.1039/d4ay00238e

[32] Prasad, R. et al. Quantifying dense multicomponent slurries with in-line ATR-FTIR and Raman spectroscopies: a Hanford case study. *Ind. Eng. Chem. Res.* **62**, 15962–15973 (2023). 37810994 10.1021/acs.iecr.3c01249 PMC10557100

[33] Mészáros, L. A. et al. Digital UV/VIS imaging: a rapid PAT tool for crushing strength, drug content and particle size distribution determination in tablets. *Int. J. Pharmaceutics* **578**, 119174 (2020). 10.1016/j.ijpharm.2020.119174 32105723

[34] Burdanova, M. G. et al. A review of the terahertz conductivity and photoconductivity of carbon nanotubes and heteronanotubes. *Adv. Opt. Mater.* **9**, 2101042 (2021).

[35] Kawabata, R. et al. Ultraflexible wireless imager integrated with organic circuits for broadband infrared thermal analysis. *Adv. Mater.* **36**, 2309864 (2024). 10.1002/adma.202309864 38213132

[36] Nakatani, M. et al. Ready-to-transfer two-dimensional materials using tunable adhesive force tapes. *Nat. Electron.* **7**, 119–130 (2024).

[37] Li, K., Suzuki, D. & Kawano, Y. Series photothermoelectric coupling between two composite materials for a freely attachable broadband imaging sheet. *Adv. Photonics Res.* **2**, 2000095 (2021).

[38] Oike, Y. Evolution of image sensor architectures with stacked device technologies. *IEEE Trans. Electron Devices* **69**, 2757–2765 (2022).

[39] Li, K. et al. All-screen-coatable photo-thermoelectric imagers for physical and thermal durability enhancement. *Adv. Mater. Interfaces* **10**, 2300528 (2023).

[40] Zhou, J. Y. et al. Room-temperature long-wave infrared detector with thin double layers of amorphous germanium and amorphous silicon. *Opt. Express* **27**, 37056–37064 (2019). 31873475 10.1364/OE.27.037056

[41] Varpula, A. et al. Nano-thermoelectric infrared bolometers. *APL Photonics* **6**, 036111 (2021).

[42] Huang, R. Z. et al. Dual-frequency CMOS terahertz detector with silicon-based plasmonic antenna. *Opt. Express* **27**, 23250–23261 (2019). 31510606 10.1364/OE.27.023250

[43] Bauer, M. et al. A high-sensitivity AlGaN/GaN HEMT terahertz detector with integrated broadband bow-tie antenna. *IEEE Trans. Terahertz Sci. Technol.* **9**, 430–444 (2019).

[44] Yang, X. X. et al. A flexible graphene terahertz detector. *Appl. Phys. Lett.* **111**, 021102 (2017).

[45] Xie, Z. M. et al. Room-ambient operation of integrated and visualized photothermoelectric system with patterned Mo_2_C/PEDOT: PSS flexible devices. *Mater. Des.* **235**, 112383 (2023).

[46] Ding, J. et al. High-performance stretchable photodetector based on CH_3_NH_3_PbI_3_ microwires and graphene. *Nanoscale* **10**, 10538–10544 (2018). 29808184 10.1039/c8nr03108h

[47] Li, K. et al. Recent progress in development of carbon-nanotube-based photo-thermoelectric sensors and their applications in ubiquitous non-destructive inspections. *Micromachines* **14**, 61 (2023). 10.3390/mi14010061 PMC9865119 36677122

[48] Li, K. et al. Simple non-destructive and 3D multi-layer visual hull reconstruction with an ultrabroadband carbon nanotubes photo-imager. *Adv. Opt. Mater.* **12**, 2302847 (2024).

[49] Fu, X. J. et al. Applications of terahertz spectroscopy in the detection and recognition of substances. *Front. Phys.* **10**, 869537 (2022).

[50] Ge, H. Y. et al. Applications of THz spectral imaging in the detection of agricultural products. *Photonics* **8**, 518 (2021).

[51] Patil, M. R. et al. Terahertz spectroscopy: encoding the discovery, instrumentation, and applications toward pharmaceutical prospectives. *Crit. Rev. Anal. Chem.* **52**, 343–355 (2022). 32772866 10.1080/10408347.2020.1802219

[52] Keysight Technologieo. Keysight 34980A multifunction switch/measure unit programmer’s reference help. at https://www.keysight.com/jp/ja/assets/9018-61230/programming-guides/9018-61230.pdf?success=true.

[53] Lu, X. W. et al. Progress of photodetectors based on the photothermoelectric effect. *Adv. Mater.* **31**, 1902044 (2019). 10.1002/adma.201902044 31483546

[54] Dong, R. Q. & Zeitler, J. A. Visualising liquid transport through coated pharmaceutical tablets using Terahertz pulsed imaging. *Int. J. Pharmaceutics* **619**, 121703 (2022). 10.1016/j.ijpharm.2022.121703 35351529

[55] Eid, S. M. et al. ATR-FTIR coupled with chemometrics for quantification of vildagliptin and metformin in pharmaceutical combinations having diverged concentration ranges. *Vibrational Spectrosc.* **106**, 102995 (2020).

[56] Zeng, F. et al. Dynamic behaviour of a conveyor belt considering non-uniform bulk material distribution for speed control. *Appl. Sci.* **10**, 4436 (2020).

[57] Li, K. et al. Robot-assisted, source-camera-coupled multi-view broadband imagers for ubiquitous sensing platform. *Nat. Commun.* **12**, 3009 (2021). 34021142 10.1038/s41467-021-23089-w PMC8139987

[58] Araki, T. et al. Broadband photodetectors and imagers in stretchable electronics packaging. *Adv. Mater.* **36**, 2304048 (2024). 10.1002/adma.202304048 37403808

[59] Suzuki, D. et al. A terahertz video camera patch sheet with an adjustable design based on self-aligned, 2D, suspended sensor array patterning. *Adv. Funct. Mater.* **31**, 2008931 (2021).

[60] Moradi, T. & Hatef, A. Thermal tracing of a highly reconfigurable and wideband infrared heat sensor based on vanadium dioxide. *J. Appl. Phys.* **127**, 243105 (2020).

[61] Aftab, S. & Hegazy, H. H. Emerging trends in 2D TMDs photodetectors and piezo-phototronic devices. *Small* **19**, 2205778 (2023). 10.1002/smll.202205778 36732842

[62] Han, M. A. & Kim, J. H. Diagnostic X-ray exposure and thyroid cancer risk: systematic review and meta-analysis. *Thyroid* **28**, 220–228 (2018). 29160170 10.1089/thy.2017.0159

