# Supplementary material for: In-line multi-wavelength non-destructive pharma quality monitoring with ultrabroadband carbon nanotubes photo-thermoelectric imaging scanners
Source: Light Sci Appl. 2025 Sep 11;14:306. doi: 10.1038/s41377-025-01957-0 (PMC12423311; doi:10.1038/s41377-025-01957-0)
Supplement: Supplementary file 1 — Supplementary Information [file 41377_2025_1957_MOESM1_ESM.pdf]

Supplementary Information for

In-line multi-wavelength non-destructive pharma quality monitoring with ultrabroadband carbon nanotubes photo-thermoelectric imaging scanners

Miki Kubota[1†], Yuya Kinoshita[1†], Sayaka Hirokawa[1], Daiki Shikichi[1], Noa Izumi[1], Naoko Hagiwara[1], Daiki Sakai[1], Yuto Matsuzaki[1], Minami Yamamoto[1], Leo Takai[1], Yukito Kon[1], Yuto Aoshima[1], Raito Ota[1], Mitsuki Kosaka[1], Meiling Sun[2,3], Yukio Kawano[1,4,5]*, and Kou Li[1]*

[1]Department of Electrical, Electronic, and Communication Engineering, Faculty of Science and Engineering, Chuo University, 1-13-27 Kasuga, Bunkyo-ku, Tokyo 112-8551, Japan

[2]Laboratory for Future Interdisciplinary Research of Science and Technology, Tokyo Institute of Technology, 2-12-1 Ookayama, Meguro-ku, Tokyo 152-8552, Japan

[3]Department of Electrical and Electronic Engineering, School of Engineering, Tokyo Institute of Technology, 2-12-1 Ookayama, Meguro-ku, Tokyo 152-8552, Japan

[4]National Institute of Informatics, 2-1-2 Hitotsubashi, Chiyoda-ku, Tokyo 101-8430, Japan

[5]Kanagawa Institute of Industrial Science and Technology, 705-1 Imaizumi, Ebina-shi, Kanagawa 243-0435, Japan

*Corresponding author: Kou Li and Yukio Kawano

†: These authors contributed equally. M. Kubota and Y. Kinoshita are co-first authors.

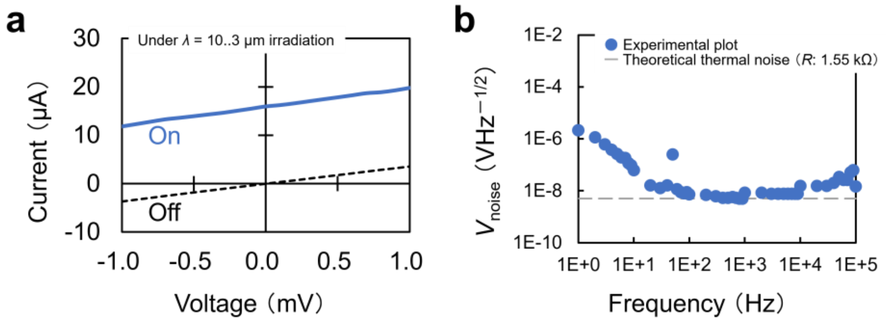

Figure S1. PTE behaviours of the CNT film imager pixel.

a, Current-voltage characteristic with and without external photo-irradiation. **b**, Noise spectrum density mapping.

Under the presented zero-voltage-bias condition, the CNT film PTE imager pixel functions at the lower limit of thermal noise signals[S1] during photo-sensing operations. Together with their uncooled experimental setups, such characteristics also design the CNT film PTE imager device as compact system configurations without employing external bulky power sources. Here in (b), the peak value at 50 Hz originates from the power source of the measurement system (not from the device itself).

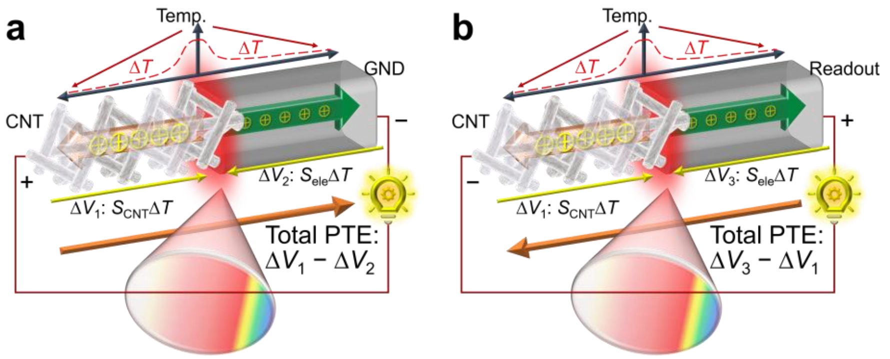

Figure S2. Channel-electrode junctions as photo-detection interfaces of the CNT film PTE imager pixel. a–b, PTE conversion models for channel-electrode junctions (w/ GND (a) and readout (b) boundaries).

Here, the respective Seebeck coefficient values for constituent materials of the PTE imager pixel are as follows: 55 μVK^{-1} for the CNT film channel and 1.5 μVK^{-1} for the wiring electrode[S2].

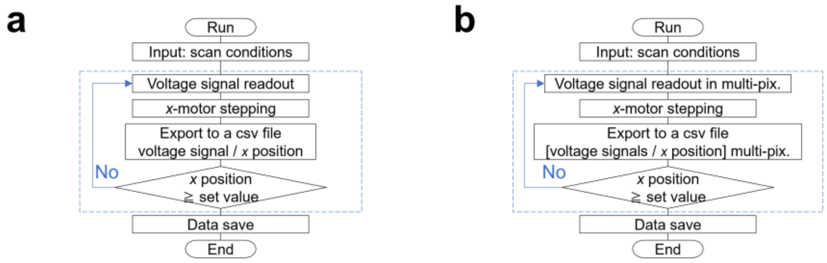

Figure S3. System configurations for spatial scanning with the CNT film PTE device.
a–**b**, System flowcharts for a single-pixel (a) and integrated imagers (b).

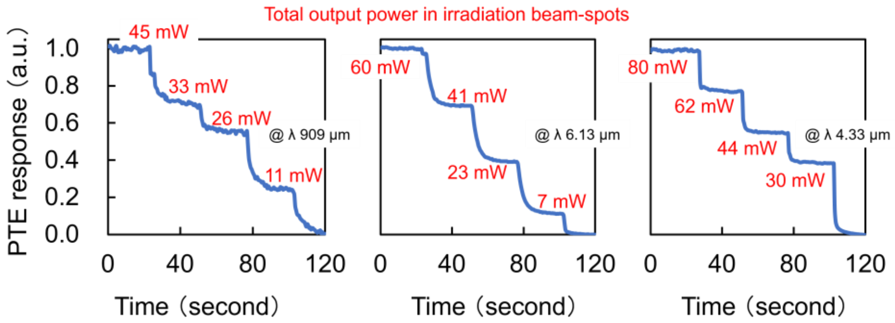

Figure S4. Changes in response signals of the CNT film PTE imager pixel per external photo-irradiation intensity.

The obtained results emphasise that the use of the CNT film PTE device facilitates acquiring monochrome images (capturing changes and gradations for transmittance values of targets) in ultrabroadband regions.

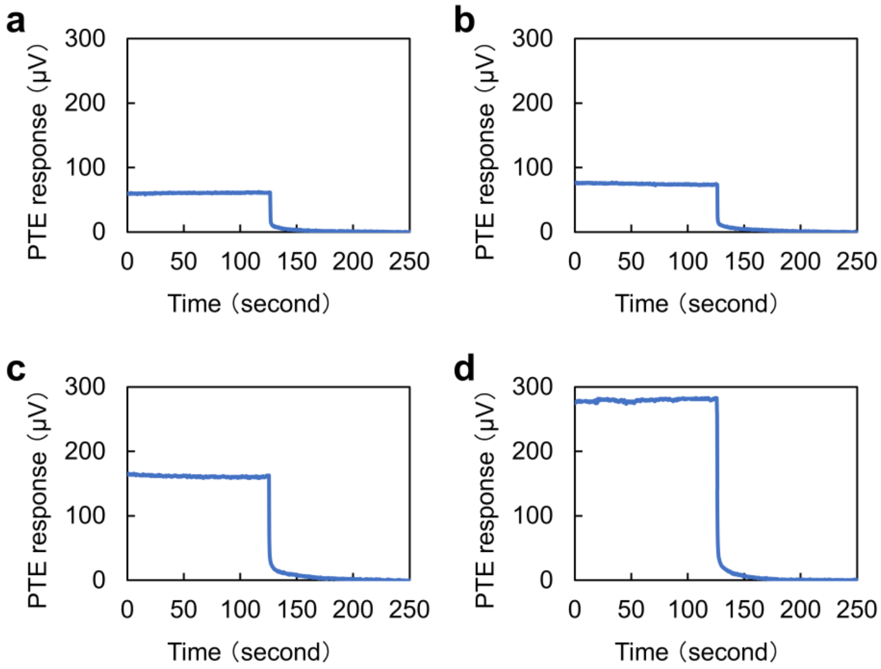

Figure S5. Wavelength dependence of photo-response signal intensities of the CNT film PTE sensor. **a**–**d**, Transient response signals of the device under external Vis (wavelength: 660 nm, a), near-IR (wavelength: 1.55 µm, b), mid-IR (wavelength: 4.33 µm, c), and far-IR (wavelength: 6.13 µm, d) irradiation.

The above evaluation unifies the photo-irradiation intensity to 8.4 mW per 2 mmφ beam-spot. The obtained result also provides a clear transient waveform with the CNT film PTE imager under on-off switching of external Vis-irradiation. This demonstration then indicates that the photo-induced PTE response signal intensity of the device increases by lengthening the external irradiation wavelength towards far-IR from Vis. This situation experimentally reflects that longer-wavelength photo-irradiation efficiently enhances heating via the lattice vibration. As the presented CNT film ultrabroadband imager functions under the PTE effect, the absorption efficiency of external irradiation and the associated photo-induced heating simultaneously govern response signal intensities of the device. Based on these trends, direct observations of photo-induced heating with the device under different wavelength photo-irradiation play essential roles in designing further sensitive ultrabroadband pharma monitoring systems based on CNT film channels as the next scope from this work.

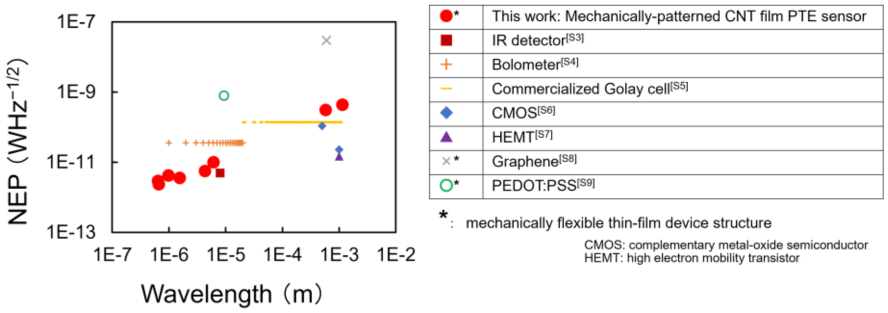

Figure S6. Operation sensitivity mapping of the representative uncooled non-vacuum photo-sensors in ultrabroadband regions.

This work employs noise equivalent power (NEP) as an index of operation sensitivity for photo-sensor devices. The following equation describes NEP as below:

$$\mathrm{NEP} = \frac{V_{\mathrm{Noise}}}{V_{\mathrm{Resopnse}}} = \frac{V_{\mathrm{Thermal\ noise}}}{S_{\mathrm{Eff}} \times \Delta T} \times P_{\mathrm{Eff}} \propto \frac{\sqrt{4k_{\mathrm{B}}TR}}{S_{\mathrm{Eff}} \times \Delta T} \qquad \text{S1}$$

where V_{Noise}, V_{Response}, $V_{\mathrm{Thermal\ noise}}$, P_{Eff}, S_{Eff}, ΔT, k_{B}, T, R are the noise voltage spectral density, normalised PTE direct-current voltage response, thermal noise voltage spectral density, effective Seebeck coefficient of CNT film-wiring electrode junctions, photo-induced temperature gradient across the channel, normalised effective output power of external irradiation onto the detection interface, absolute temperature, electrical resistance of the device, respectively[S10].

Table S1. Fundamental characteristics of the representative uncooled non-vacuum photo-sensors.

*CMOS: Complementary Metal Oxide Semiconductor, HEMT: High Electron Mobility Transistor, CEP: Cycling Excitation Process, a-Ge: amorphous germanium, a-Si: amorphous silicon, TiW: Titanium-Tungsten, TiN: tungsten nitride, AlGaN/GaN: aluminum-gallium nitride/gallium nitride, Mo_2C: Molybdenum Carbide

Type (Ref.)	Mechanism	Frequency	Material	Min. NEP	Structure / Resolution
This work	PTE effect	*Ultrabroad* MMW–Vis	CNT film	1.2 $pWHz^{-1/2}$	Freely designable (Stretchable thin-sheets, 3D modules, etc) Res.: 400 μm
IR sensor (S3)	Photo-conductive, CEP	MIR	a-Ge, a-Si	5 $pWHz^{-1/2}$	Rigid solid-state Res.: Single-pixel
Bolometers (S4)	Temperature -induced current	*Broad* IR	TiW, TiN	36 $pWHz^{-1/2}$	Rigid solid-state Res.: 170 μm
CMOS (S6)	High-freq. circuit	*Broad* THz	Silicon, Metal	23 $pWHz^{-1/2}$	Rigid solid-state Res.: Single-pixel
HEMT (S7)	High-freq. circuit	MMW	AlGaN/GaN	15 $pWHz^{-1/2}$	Rigid solid-state Res.: Single-pixel
Graphene (S8)	Plasmonic	Sub-THz	Graphene	3 $nWHz^{-1/2}$	Flexible sheet Res.: Single-pixel
PEDOT:PSS (S9)	PTE effect	FIR	Mo_2C/PEDOT :PSS	800 $pWHz^{-1/2}$	Flexible sheets Res.: Single-pixel
Microwire (S11)	Photodiode	*Broad* NIR–UV	Perovskite, Graphene	N.A.	Stretchable sheets Res.: Single-pixel

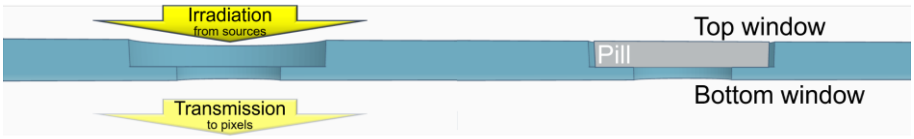

Figure S7. Sectional views of the sample holder for in-line pill conveyor operations in this work.

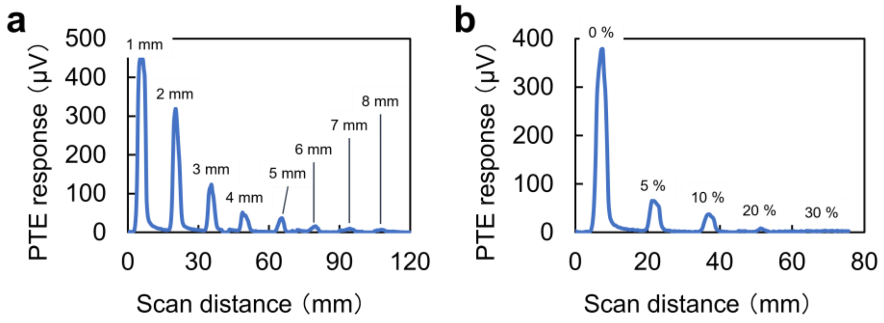

Figure S8. Available configurations of pharma agent pills for the presented in-line monitoring system in this work.

a–**b**, Changes in PTE responses of the CNT film PTE imager pixel per pill thickness (a) and concentration (b) for in-line monitoring.

This measurement refers to responses of the middle pixel (centre position) of the imager array under external SWIR-irradiation. Targeted pills (set within the conveyor jig (Figure S7)) consequently and perpendicularly move through optical paths of the presented in-line system. This work employs "Sedative" for (a) and "Antipyretic" for (b).

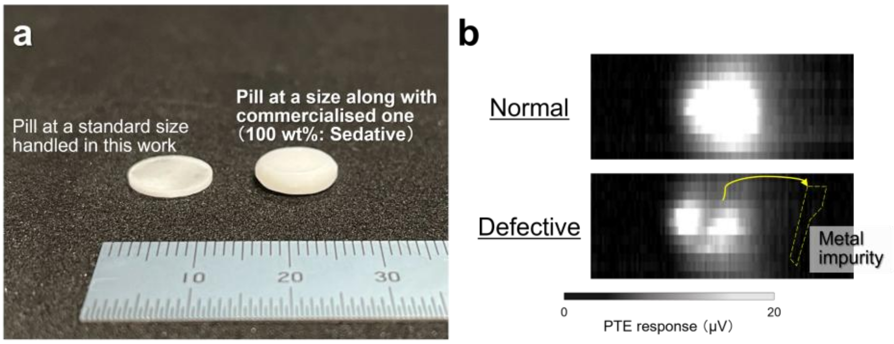

Figure S9. Non-destructive monitoring with the CNT film PTE imager of practical pharma pills. **a**, Photograph of a real-scale sedative pill. **b**, SWIR monitoring of the real-scale pills with the CNT film PTE imager.

The obtained results experimentally emphasise that the presented CNT film PTE imager-based non-destructive pharma monitoring system is even suitable for real-scale pills, by properly extracting a concealed hazardous metal impurity. As practical manufacturing situations of pharmaceutical products include not only inner pills themselves but also outer containers (e.g., blister- and press-through-packages), one of the essential next scopes derived from this work is an in-line dynamic demonstration of non-destructive multi-scale monitoring for entire assembled elements with CNT film PTE imagers under external ultrabroadband photo-irradiation.

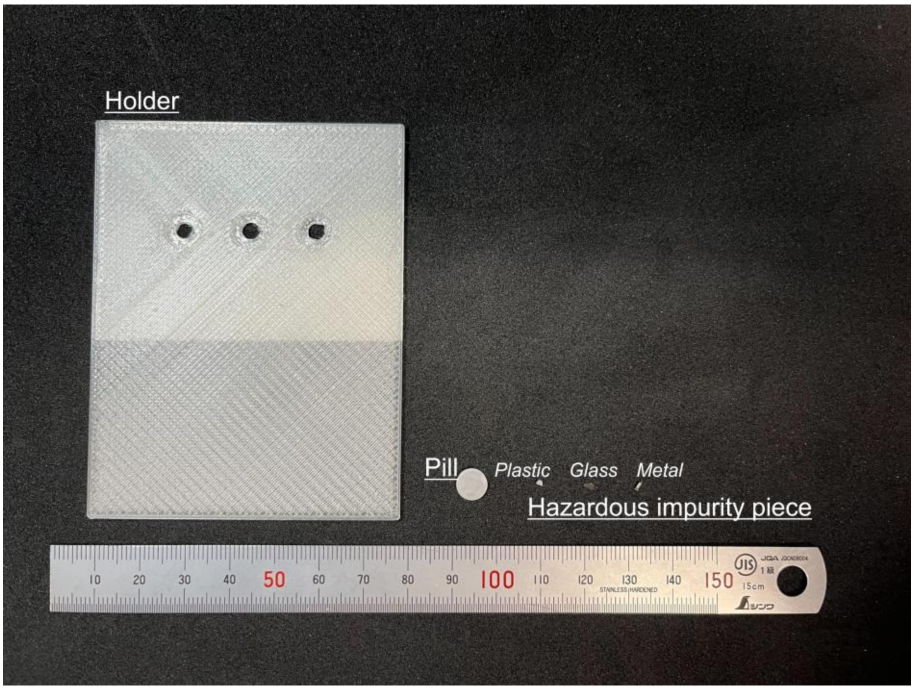

Figure S10. Sample preparation for non-destructive in-line dynamic monitoring of pharma agent pills with hazardous impurity pieces concealed inside of them.

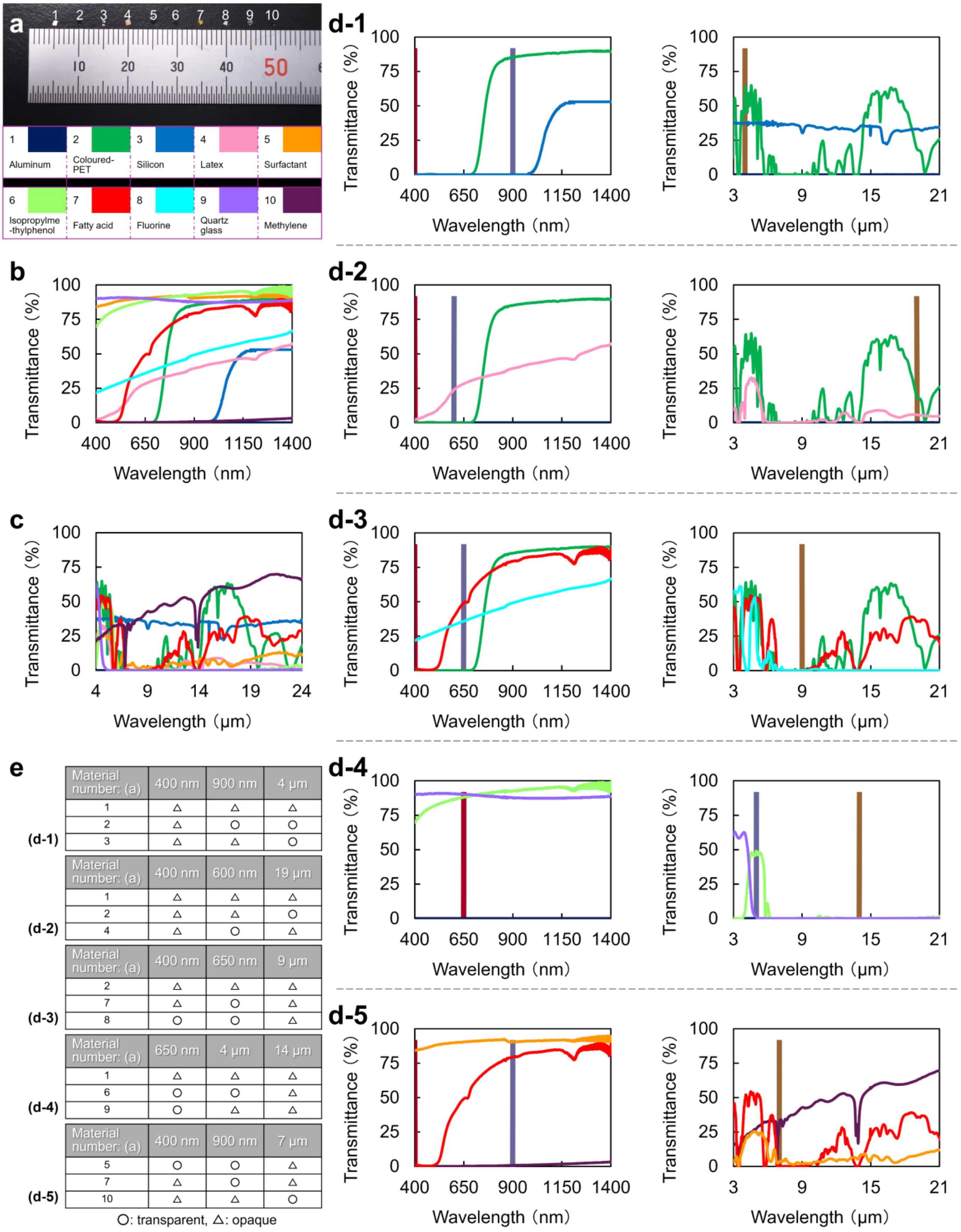

(d-1) Material number: (a)	400 nm	900 nm	4 μm
1	△	△	△
2	△	○	○
3	△	△	○

(d-2) Material number: (a)	400 nm	600 nm	19 μm
1	△	△	△
2	△	△	○
4	△	○	△

(d-3) Material number: (a)	400 nm	650 nm	9 μm
2	△	△	△
7	△	○	△
8	○	○	△

(d-4) Material number: (a)	650 nm	4 μm	14 μm
1	△	△	△
6	○	○	△
9	○	△	△

(d-5) Material number: (a)	400 nm	900 nm	7 μm
5	○	○	△
7	△	○	△
10	△	△	○

○: transparent, △: opaque

Figure S11. Database for potential non-destructive material composition identification of concealed impurities in pharma assembling processes under external broadband photo-irradiation.

a, Tested impurities. The colour-assignment for respective impurities corresponds to the following waveforms (b–d). **b**–**c**, Fundamental optical properties of respective impurities (a) via UV-Vis-NIR (b) and FTIR (c)

spectroscopy. **d**, Potential combinations for non-destructive material composition identification of impurities (a) via broadband three-wavelength photo-monitoring. **e**, Diagrams in (d).

The obtained result systematically handles 10 different types of materials as hazardous impurities in pharma pills (a). The above demonstration first summarises the fundamental spectroscopic behaviours of respective impurities, which effectively serve as the key information source to organise the presented experimental setup of broadband multi-wavelength in-line pharma monitoring in this work (b,c). Based on these preparations, (d) introduces potential combinations for non-destructive material composition identification of impurities via broadband three-wavelength photo-monitoring, along with conditions of the experimental demonstrations achieved in this work. The vertical colour bars respectively correspond to the three photo-irradiation wavelengths, supposed to be extracted for in-line experimental setups, as follows: dark red (shortest), dark blue (middle), and ochre (longest). Figure S11e further breaks down the above potential combinations for non-destructive impurity identification with the presented device and system. For example, a diagram chart "d-4" indicates that three-wavelength photo-monitoring at 650 nm, 4 μm, and 14 μm regions results in non-destructive identification among "Aluminium", "Isopropylmethylphenol", and "Quartz glass". By incorporating the presented automatic classification with thresholds in response signal intensities of CNT film PTE imagers (Supplementary Movie 2) and the above pre-performed database, the system in this work potentially provides high universality to broader operators as non-destructive in-line dynamic pharma monitoring tools.

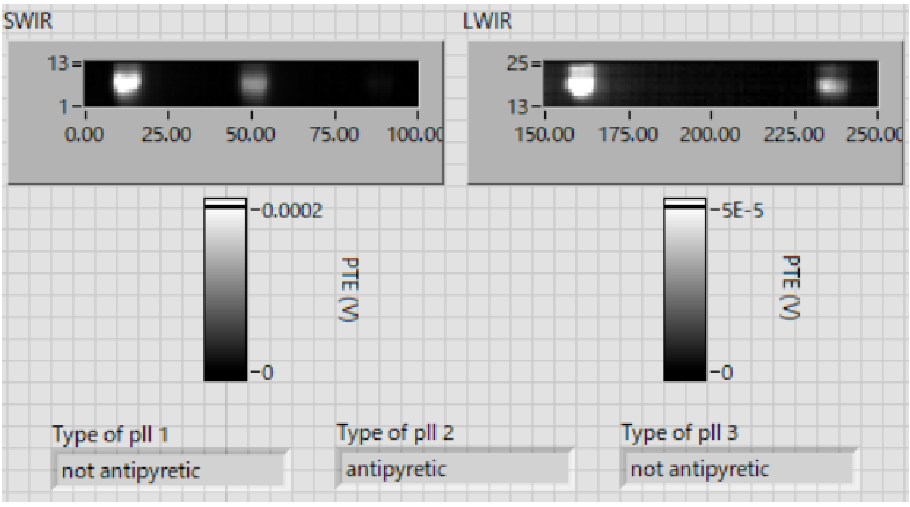

Figure S12. Thumbnail of Supplementary Movie 3.

Along Figure 5b, this work also applies threshold transmittance values of different type pharma agent pills under external multi-wavelength IR-irradiation for exception composition monitoring with the CNT film PTE imager in a non-destructive in-line dynamic manner.

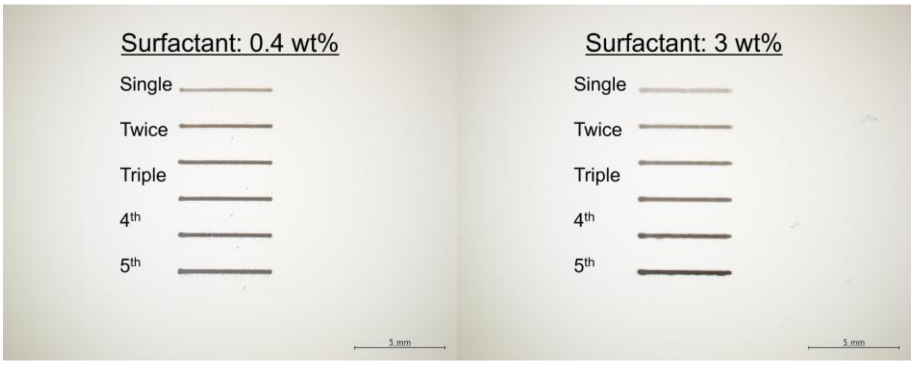

Figure S13. Photographs of CNT film channels with different in-liquid surfactant ratios for mechanically alignable air-jet dispense-printing processes.

In respective photographs, the mentioned number count corresponds to repetitive printing cycles of CNT inks. Each electrical resistance value per CNT composition and printing cycle is as follows: 0.4 wt% (660 Ω for single, 332 Ω for twice, 222 Ω for triple, 154 Ω for 4th, and 125 Ω for 5th) and 3 wt% (10.8 kΩ for single, 4.16 kΩ for twice, 2.26 kΩ for triple, 1.68 kΩ for 4th, and 1.55 kΩ for 5th).

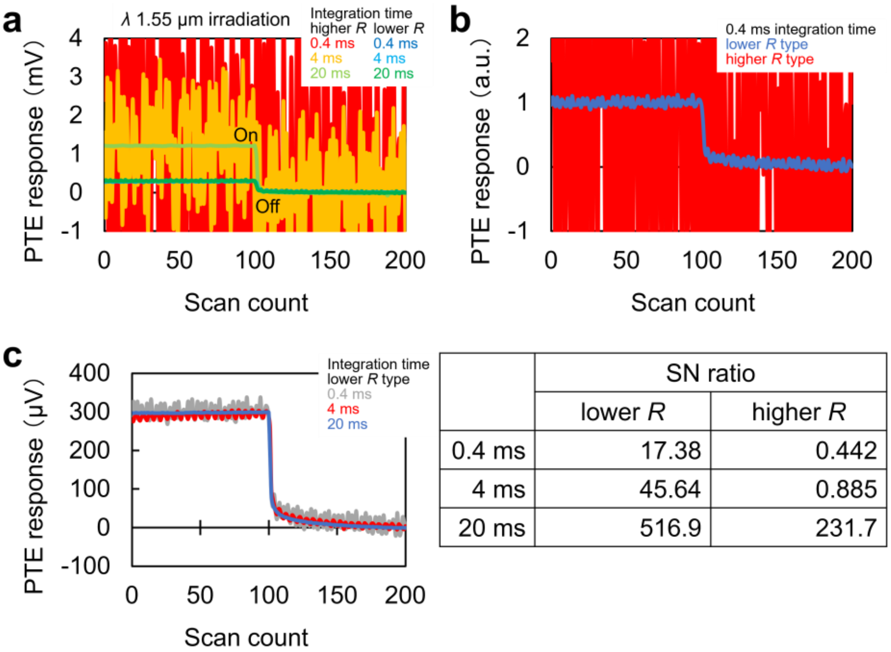

	SN ratio	
	lower *R*	higher *R*
0.4 ms	17.38	0.442
4 ms	45.64	0.885
20 ms	516.9	231.7

Figure S14. Comparisons of photo-detection behaviours of CNT film PTE imager pixels with different in-liquid surfactant ratios under single solution printing.

a, Changes in PTE responses of CNT film PTE imager pixels with different integration time conditions in the employed datalogger. **b**, Change in PTE responses of CNT film PTE imager pixels at the fastest integration time among the employed experimental conditions in this work. **c**, Change in PTE responses of the CNT film PTE imager pixel (in-liquid surfactant ratio: 0.4 wt%) with different integration time conditions in the employed datalogger.

In the figure index, “lower *R*” and “higher *R*” respectively correspond to left-hand-side and right-hand-side CNT compositions in Figure S13. The main text of this manuscript describes interrelations between datalogger configurations (integration time conditions and the number of power line cycle values). A series of the obtained result (Figure S14–S18) infers that the use of the CNT film PTE imager pixel with the lower in-liquid surfactant ratio leads to 4.86–39.3 times sensitivity enhancements than that with the higher one at the fastest signal readout speed in photo-detection operations. Although repetitive printing cycles of CNT solutions with the higher in-liquid surfactant ratio suppress crucial noise signals in photo-detection operations (as shown in Figure S18), such approaches subsequently complicate fabrication processes of imager devices and potentially induce unnecessary blurriness in line widths. To maintain the simplicity in fabrications processes and printing yields in multiple-pixels integrations, the presenting ultrabroadband PTE imager device in this work employs the CNT film (in-liquid surfactant ratio: 0.4 wt%) as its major constituent material. For calculating signal-to-noise (SN) ratios shown in the figure, this work refers to root mean square values of error ratios in photo-detection response signals. λ: 1.55 µm (b–c).

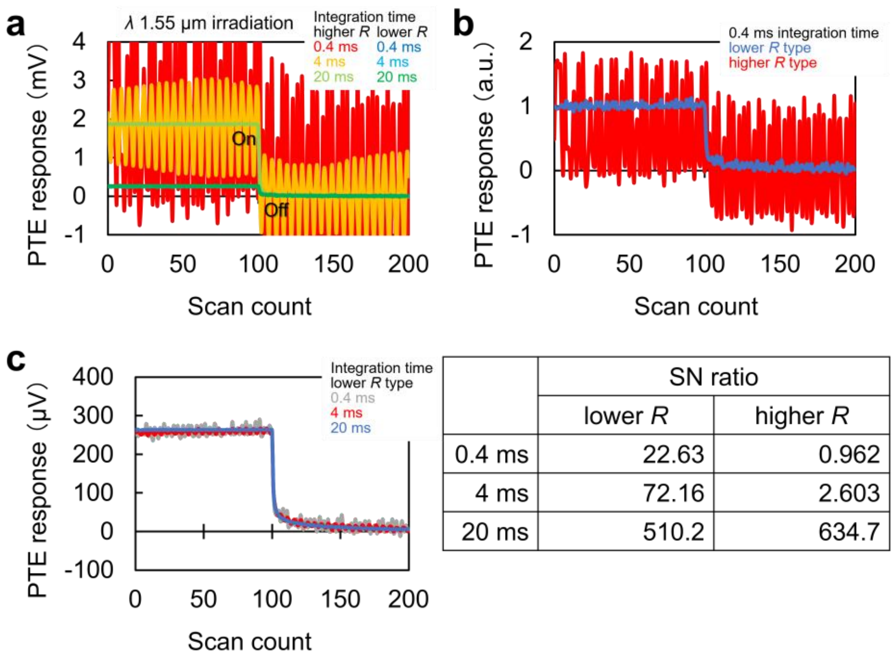

	SN ratio	
	lower *R*	higher *R*
0.4 ms	22.63	0.962
4 ms	72.16	2.603
20 ms	510.2	634.7

Figure S15. Comparisons of photo-detection behaviours of CNT film PTE imager pixels with different in-liquid surfactant ratios under twice solution printing.

a, Changes in PTE responses of CNT film PTE imager pixels with different integration time conditions in the employed datalogger. **b**, Change in PTE responses of CNT film PTE imager pixels at the fastest integration time among the employed experimental conditions in this work. **c**, Change in PTE responses of the CNT film PTE imager pixel (in-liquid surfactant ratio: 0.4 wt%) with different integration time conditions in the employed datalogger.

In the figure index, "lower *R*" and "higher *R*" respectively correspond to left-hand-side and right-hand-side CNT compositions in Figure S13. The main text of this manuscript describes interrelations between datalogger configurations (integration time conditions and the number of power line cycle values). For calculating signal-to-noise (SN) ratios shown in the figure, this work refers to root mean square values of error ratios in photo-detection response signals. λ: 1.55 μm (b–c).

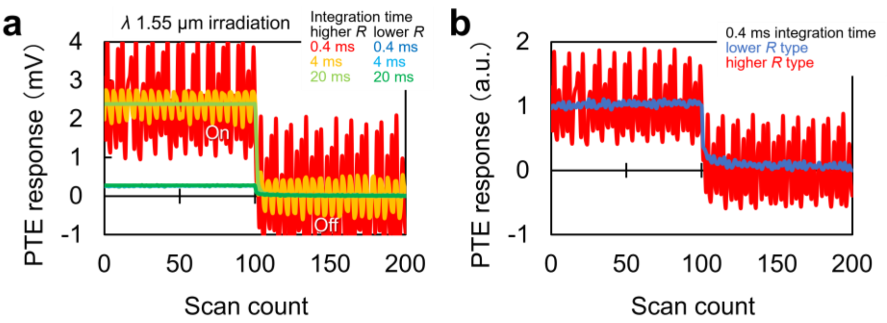

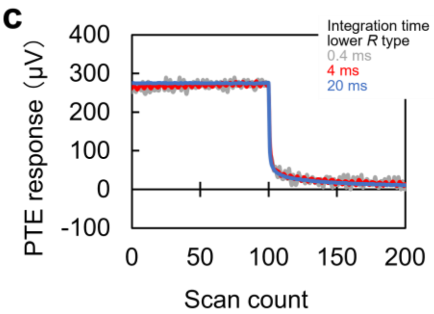

	SN ratio	
	lower *R*	higher *R*
0.4 ms	23.56	2.335
4 ms	67.53	8.247
20 ms	453.2	1092

Figure S16. Comparisons of photo-detection behaviours of CNT film PTE imager pixels with different in-liquid surfactant ratios under triple solution printing.

a, Changes in PTE responses of CNT film PTE imager pixels with different integration time conditions in the employed datalogger. **b**, Change in PTE responses of CNT film PTE imager pixels at the fastest integration time among the employed experimental conditions in this work. **c**, Change in PTE responses of the CNT film PTE imager pixel (in-liquid surfactant ratio: 0.4 wt%) with different integration time conditions in the employed datalogger.

In the figure index, "lower *R*" and "higher *R*" respectively correspond to left-hand-side and right-hand-side CNT compositions in Figure S13. The main text of this manuscript describes interrelations between datalogger configurations (integration time conditions and the number of power line cycle values). For calculating signal-to-noise (SN) ratios shown in the figure, this work refers to root mean square values of error ratios in photo-detection response signals. λ: 1.55 μm (b–c).

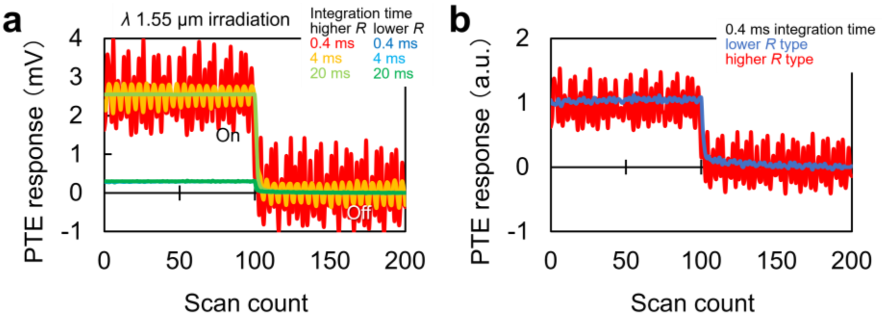

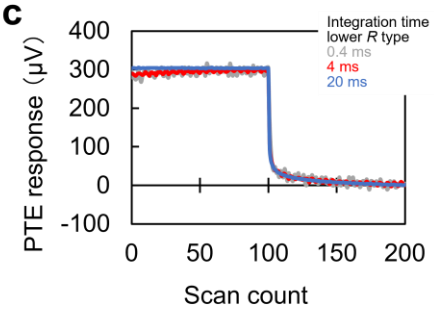

	SN ratio	
	lower *R*	higher *R*
0.4 ms	32.79	3.625
4 ms	74.03	10.07
20 ms	589.9	1161

Figure S17. Comparisons of photo-detection behaviours of CNT film PTE imager pixels with different in-liquid surfactant ratios under 4th solution printing.

a, Changes in PTE responses of CNT film PTE imager pixels with different integration time conditions in the employed datalogger. **b**, Change in PTE responses of CNT film PTE imager pixels at the fastest integration time among the employed experimental conditions in this work. **c**, Change in PTE responses of the CNT film PTE imager pixel (in-liquid surfactant ratio: 0.4 wt%) with different integration time conditions in the employed datalogger.

In the figure index, “lower *R*” and “higher *R*” respectively correspond to left-hand-side and right-hand-side CNT compositions in Figure S13. The main text of this manuscript describes interrelations between datalogger configurations (integration time conditions and the number of power line cycle values). For calculating signal-to-noise (SN) ratios shown in the figure, this work refers to root mean square values of error ratios in photo-detection response signals. λ: 1.55 μm (b–c).

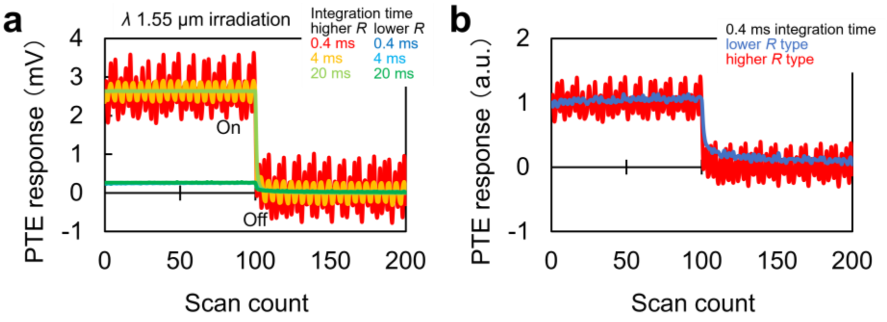

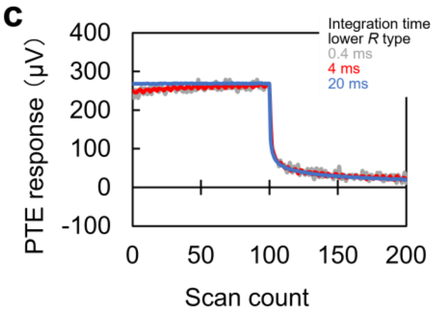

	SN ratio	
	lower *R*	higher *R*
0.4 ms	24.76	5.090
4 ms	51.09	13.65
20 ms	450.3	1840

Figure S18. Comparisons of photo-detection behaviours of CNT film PTE imager pixels with different in-liquid surfactant ratios under 5th solution printing.

a, Changes in PTE responses of CNT film PTE imager pixels with different integration time conditions in the employed datalogger. **b**, Change in PTE responses of CNT film PTE imager pixels at the fastest integration time among the employed experimental conditions in this work. **c**, Change in PTE responses of the CNT film PTE imager pixel (in-liquid surfactant ratio: 0.4 wt%) with different integration time conditions in the employed datalogger.

In the figure index, "lower *R*" and "higher *R*" respectively correspond to left-hand-side and right-hand-side CNT compositions in Figure S13. The main text of this manuscript describes interrelations between datalogger configurations (integration time conditions and the number of power line cycle values). For calculating signal-to-noise (SN) ratios shown in the figure, this work refers to root mean square values of error ratios in photo-detection response signals. λ: 1.55 µm (b–c).

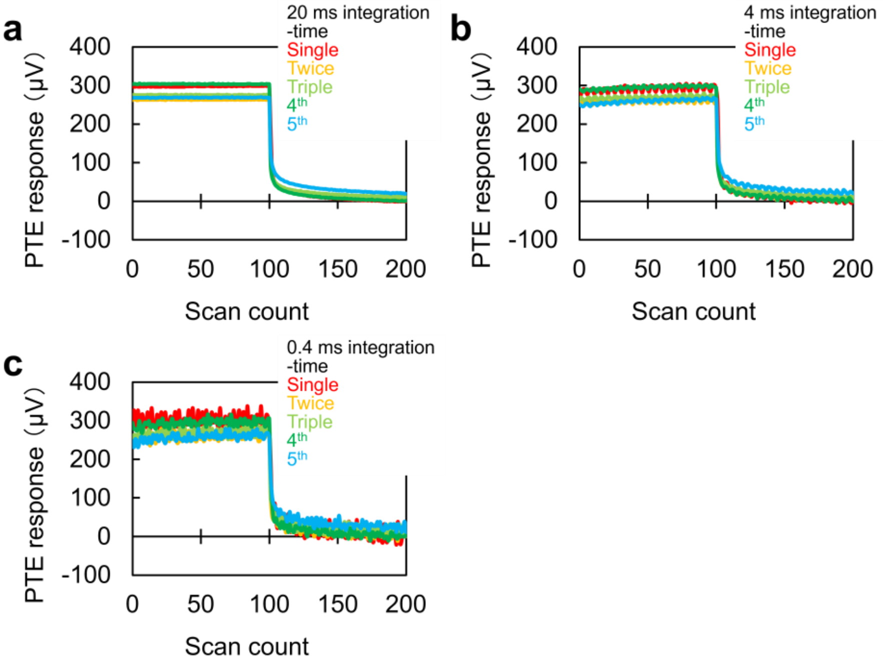

Figure S19. Comparisons of photo-detection behaviours of the CNT film PTE imager pixels (in-liquid surfactant ratio: 0.4 wt%) per repetitive constituent material solution printing cycle and integration time condition of the employed datalogger.

a–**c**, Changes in PTE response of the CNT film imager pixels under on-off switching operations of external photo-irradiation for respective 20 ms (a), 4 ms (b), and 0.4 ms (c) integration time conditions.

The main text of this manuscript describes interrelations between datalogger configurations (integration time conditions and the number of power line cycle values). λ: 1.55 μm (a–c).

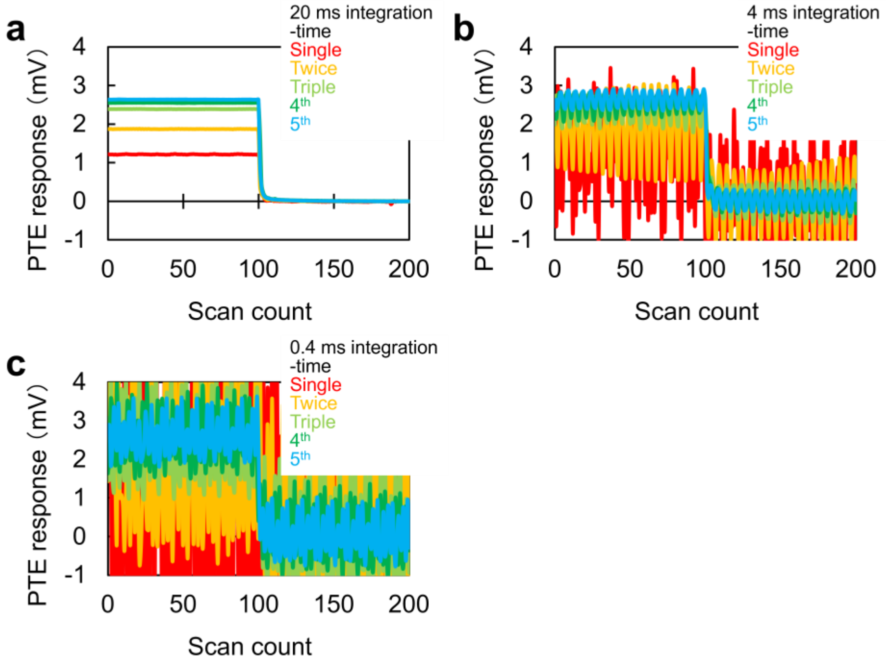

Figure S20. Comparisons of photo-detection behaviours of the CNT film PTE imager pixels (in-liquid surfactant ratio: 3 wt%) per repetitive constituent material solution printing cycle and integration time condition of the employed datalogger.

a–**c**, Changes in PTE response of the CNT film imager pixels under on-off switching operations of external photo-irradiation for respective 20 ms (a), 4 ms (b), and 0.4 ms (c) integration time conditions.

The main text of this manuscript describes interrelations between datalogger configurations (integration time conditions and the number of power line cycle values). λ: 1.55 μm (a–c).

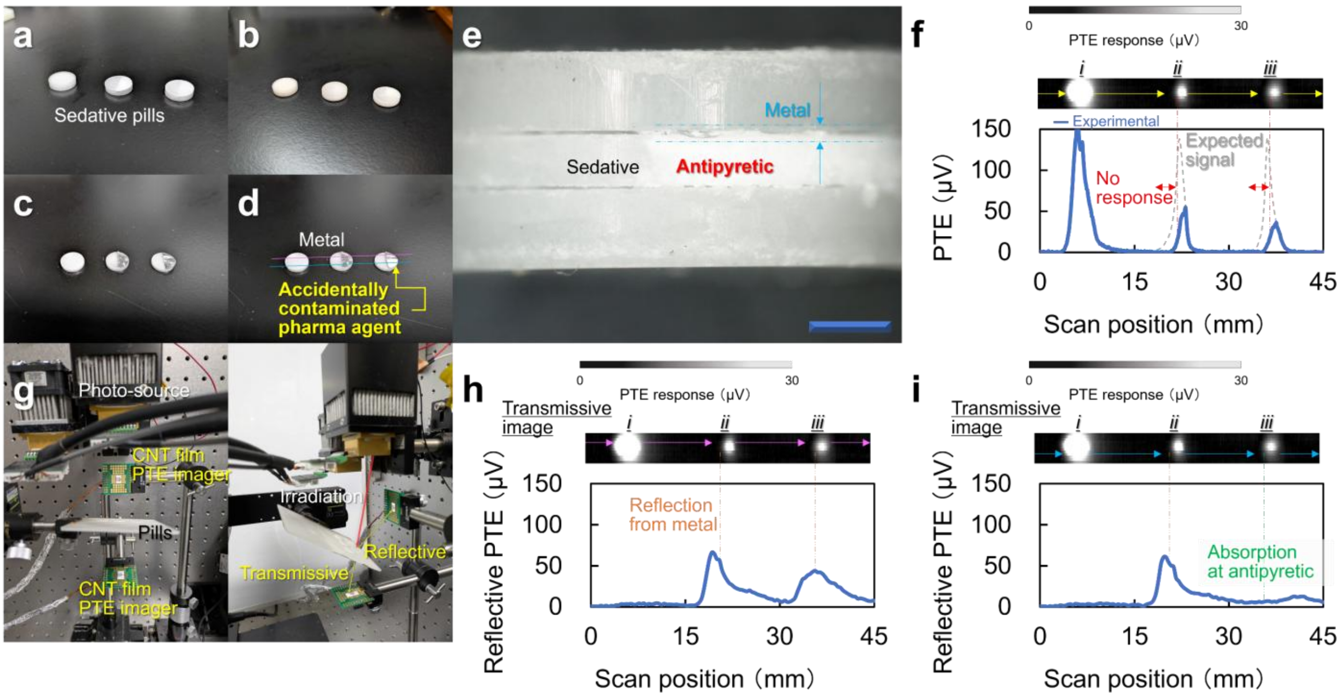

Figure S21. Hybrid optical system (transmissive/reflective) for pharma quality monitoring of samples including metal impurities.

a–**e**, Photographs of tested pharma pill samples from the viewpoint of front (a), back (b), front (*disassembled, c), back (*disassembled, d), and cross-sectional (*optical-microscope for the right-hand-side one in (a–d), e). Scale-bar: 2 mm. **f**, Transmissive SWIR monitoring of samples (a–e) by the CNT film PTE imager. **g**, Experimental setups for the hybrid pharma monitoring. **h**–**i**, Reflective line-profiling of samples (a–e) by the device at pink-coloured (h) and light blue-coloured (i) positions under SWIR-irradiation.

For the tested pharma sedative pills, two samples at spatial positions of the centre and right-hand-side in the photographs (a–d) are defective. Both pills conceal semicircle-shaped metal impurities inside as invisible defects, and the right-hand-side one further contains a quadrant-shaped different pharma agent (antipyretic).

The obtained result experimentally demonstrates the significance of a reflective optical system in performing detailed evaluations of metallic impurities concealed in pharma pills. The inherent reflection at metals against external photo-irradiation and the associated changes in their signal intensities communicate the fundamental surface characteristics. In this demonstration, the use of transmissive optical systems (along with major measurements in this work) with CNT film PTE imagers already notices the existence of semicircle-shaped metal impurities concealed in two sedative pills (transparent under SWIR-irradiation) by visualising their shapes in obtained views. However, this case study further conceals a different pharma agent "antipyretic (opaque under SWIR-irradiation)" on the metal impurity as a quadrant shape within a sedative pill, and the CNT film PTE imager-based transmissive view is insufficient for non-destructively identifying the above defect. Against this situation, (g–i) selectively extracts the existence of the accidentally contaminated antipyretic agent in the opaque sedative pill via reflective SWIR line-profiling with the CNT film PTE imager in a non-destructive manner. By incorporating these techniques and findings in this demonstration, further hybrid optical setups with CNT film PTE imagers potentially enrich the universality of the presented non-destructive in-line dynamic pharma monitoring system against diverse products.

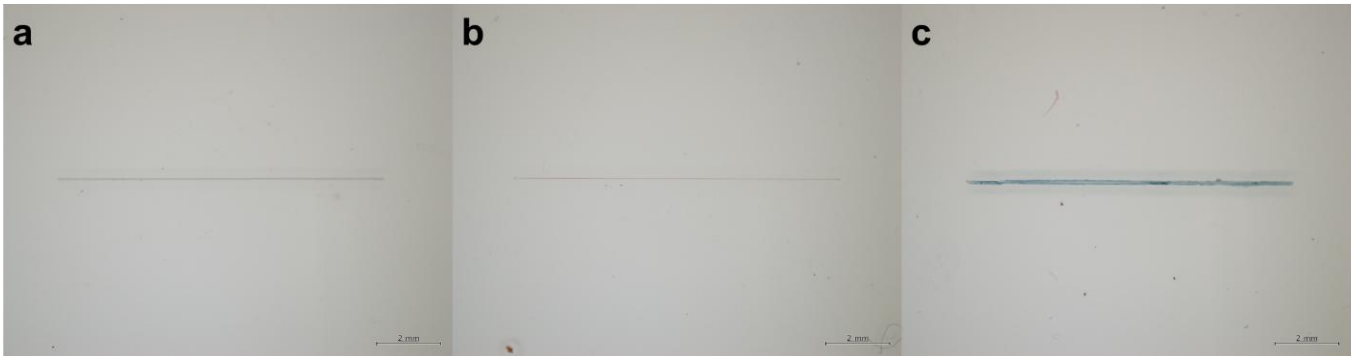

Figure S22. Electric field-driven inkjet-printing of potential constituent materials for PTE imagers at ultrahigh spatial resolution.

a–**c**, Optical-microscope images of inkjet-printed channel lines by CNT (a), silver-paste (b), and conductive polymer (c).

This work employs (Super Inkjet Printer, SIJ Technology Inc.) for the fabrication equipment, and (Poly(3,4-ethylenedioxythiophene)poly(styrenesulfonate), 739316, Sigma Aldrich Co.) for the conductive polymer. The measured line widths of respective inkjet-printed channels are as follows: 46.1 μm (a), 9.90 μm (b), and 158 μm (c).

The obtained result experimentally emphasises that the use of an electric field-driven inkjet-printer facilitates the development of high spatial resolution CNT film PTE imagers. While the presented air-jet dispense-printing results in the integration of CNT film PTE pixels at a pitch of 400 μm with respective channel line widths of 300 μm. The above demonstration indeed provides line widths of 46.1 μm and 9.90 μm for an inkjet-printed CNT film channel and a silver-electrode. Although CNTs-unique in-liquid bundles at a size order of micrometres[S12] within aqueous dispersions are crucial bottlenecks as nozzle clogging towards high-yield inkjet-printing, recent advances in the study field of the nanometre-scale mechanical homogenisation[S13] potentially relax such technical challenges. As the above demonstration confirms the inkjet-printed electrode line width within 10 μm at this moment, the next scope from this work regarding precise homogenisation approaches of CNT inks plays a key role in developing CNT film PTE imagers with a few μm spatial resolution for more reliable non-destructive pharma monitoring applications. To tackle this outlook, the fundamental material science (e.g., PTE properties of inkjet-printed CNT films w/ and w/o precise homogenisation) further facilitates a deeper understanding of the presented device and system towards widely available pharma monitoring platforms. Note that static chemical analysers at early drug discovery stages prior to in-line pharmaceutical manufacturing phases (requiring full evaluation via compact modules such as the presented device and system in this work) provides a spatial resolution of 2 μm in impurity monitoring[S14].

Supplementary References

S1. Li, K. et al. Stretchable broadband photo-sensor sheets for nonsampling, source-free, and label-free chemical monitoring by simple deformable wrapping. *Sci. Adv.* **8**, eabm4349 (2022).

S2. Li, K. et al. Robot-assisted, source-camera-coupled multi-view broadband imagers for ubiquitous sensing platform. *Nat. Commun.* **12**, 3009 (2021).

S3. Zhou, J. et al. Room-temperature long-wave infrared detector with thin double layers of amorphous germanium and amorphous silicon. *Opt. Express* **27**, 37056–37064 (2019).

S4. Varpula, A. et al. Nano-thermoelectric infrared bolometers. *APL Photon.* **6**, 036111 (2021).

S5. TYDEX LLC. https://www.tydexoptics.com/pdf/Golay_Detectors.pdf view date: 18th September, 2024.

S6. Huang, R. et al. Dual-frequency CMOS terahertz detector with silicon-based plasmonic antenna. *Opt. Express* **27**, 23250–23261 (2019).

S7. Bauer, M. et al. A High-Sensitivity AlGaN/GaN HEMT Terahertz Detector With Integrated Broadband Bow-Tie Antenna. *IEEE Trans. Terahertz Sci. Technol.* **9**, 430–444 (2019).

S8. Yang, X., Vorobiev, A., Generalov, A., Andersson, M. A. & Stake, J. A flexible graphene terahertz detector. *Appl. Phys. Lett.* **111**, 021102 (2017).

S9. Xie, Z., Wang, J., Lu, G. & Yeow, J. T. W. Room-ambient operation of integrated and visualized photothermoelectric system with patterned Mo_2C/PEDOT: PSS flexible devices. *Mater. Des.* **235**, 112383 (2023).

S10. Li. K., Suzuki. D. & Kawano, Y. Series Photothermoelectric Coupling Between Two Composite Materials for a Freely Attachable Broadband Imaging Sheet. *Adv. Photon. Res.* **2**, 2000095 (2021).

S11. Ding, J. et al. High-performance stretchable photodetector based on $CH_3NH_3PbI_3$ microwires and graphene. *Nanoscale* **10**, 10538–10544 (2018).

S12. Nomura, A. et al. M. Highly-porous Super-Growth carbon nanotube sheet cathode develops high-power Lithium-Air Batteries. *Electrochim. Acta* **400**, 139415 (2021).

S13. Yadav, P., Gupta, S. M. & Sharma S. K. A review on stabilization of carbon nanotube nanofluid. *J. Therm. Anal. Calorim.* **147**, 6537–6561 (2022).

S14. Hiemenz, C. et al. Characterization of Virus Particles and Submicron-Sized Particulate Impurities in Recombinant Adeno-Associated Virus Drug Product. *J. Pharm. Sci.* **112**, 2190–2202 (2023).
